# Optimization and Anti-Cancer Properties of Fluoromethylketones as Covalent Inhibitors for Ubiquitin C-Terminal Hydrolase L1

**DOI:** 10.3390/molecules26051227

**Published:** 2021-02-25

**Authors:** Aaron D. Krabill, Hao Chen, Sajjad Hussain, Chad S. Hewitt, Ryan D. Imhoff, Christine S. Muli, Chittaranjan Das, Paul J. Galardy, Michael K. Wendt, Daniel P. Flaherty

**Affiliations:** 1 Department of Medicinal Chemistry and Molecular Pharmacology, College of Pharmacy, 575 Stadium Mall Dr., West Lafayette, IN 47907, USA; akrabil@purdue.edu (A.D.K.); chen2334@purdue.edu (H.C.); hewitt8@purdue.edu (C.S.H.); rimhoff@purdue.edu (R.D.I.); cmuli@purdue.edu (C.S.M.); mwendt@purdue.edu (M.K.W.); 2 Division of Pediatric Hematology-Oncology, Mayo Clinic, 200 First St. Guggenheim 15, Rochester, MN 55905, USA; hussain.sajjad@mayo.edu (S.H.); galardy.paul@mayo.edu (P.J.G.); 3 Department of Pediatric and Adolescent Medicine, Mayo Clinic, 200 First St. Guggenheim 15, Rochester, MN 55905, USA; 4 Department of Chemistry, College of Science, 560 Oval Dr., West Lafayette, IN 47907, USA; cdas@purdue.edu; 5 Purdue Center for Cancer Research, Hanson Life Sciences Research Building, 201 University St., West Lafayette, IN 47907, USA; 6 Purdue Institute for Drug Discovery, 720 Clinic Ln., West Lafayette, IN 47907, USA

**Keywords:** covalent inhibitors, fluoromethylketones, UCHL1 inhibitors, deubiquitinases

## Abstract

The deubiquitinating enzyme (DUB) UCHL1 is implicated in various disease states including neurodegenerative disease and cancer. However, there is a lack of quality probe molecules to gain a better understanding on UCHL1 biology. To this end a study was carried out to fully characterize and optimize the irreversible covalent UCHL1 inhibitor VAEFMK. Structure-activity relationship studies identified modifications to improve activity versus the target and a full cellular characterization was carried out for the first time with this scaffold. The studies produced a new inhibitor, **34**, with an IC_50_ value of 7.7 µM against UCHL1 and no observable activity versus the closest related DUB UCHL3. The molecule was also capable of selectively inhibiting UCHL1 in cells and did not demonstrate any discernible off-target toxicity. Finally, the molecule was used for initial probe studies to assess the role of UCHL1 role in proliferation of myeloma cells and migration behavior in small cell lung cancer cells making **34** a new tool to be used in the biological evaluation of UCHL1.

## 1. Introduction

Ubiquitination of substrate proteins plays a role in a number of cellular pathways including protein trafficking, DNA damage response, and proteasomal degradation [1,2,3]. The formation of a covalent bond between the C-terminal carboxylate of ubiquitin (Ub) and the primary amine on a lysine side chain of substrate proteins results in an isopeptide bond. This bond formation is catalyzed by a series of cascading E1 (Ub-activating), E2 (Ub-conjugating), and E3 (Ub-ligating) enzymes, which often results in the addition of Ub in the form of a poly-Ub chain in both linear and branched architectures [4,5,6,7,8,9]. The identity of these Ub-chains forms the basis of the complex Ub-signaling pathway observed in eukaryotes.

The removal of Ub is catalyzed by deubiquitinases (DUBs), of which there are approximately 100 in the human genome [10]. The majority of these DUBs are cysteine proteases, although a family of DUBs, the JAMM proteases, do require a zinc cofactor for catalysis [7]. The cysteine protease DUBs are categorized into six sub-families: ubiquitin specific proteases (USP), Machado-Josephin domain (MJD), Ovarian tumor proteases (OTU), Ubiquitin C-terminal hydrolases (UCH), and the recently discovered MINDY and ZUFSP DUBs [11,12]. Dysregulation of protein ubiquitination status is implicated in a number of disease states including cancer [13,14,15] and neurodegeneration [16,17], and as such DUBs have emerged as potential therapeutic targets [18,19,20].

Ubiquitin C-terminal Hydrolase L1 (UCHL1) is a 25 kDa DUB whose ectopic expression is linked to a number of cancers [21]. Normally found in the central and peripheral nervous system, its physiological role under normal conditions is not well understood [22]. UCHL1 comprises approximately 1–5% of the total soluble protein in the brain [23] and is implicated in the progression of neurodegenerative diseases [24,25,26,27]. It is also considered an oncogene in a number of cancers including lymphoma [28,29] small-cell lung cancer [30,31,32], glioblastoma [33], and myeloma [34,35,36] among others [37,38]. Additionally, expression of UCHL1 in these cancers often correlates with increased metastatic behavior and poor patient prognosis [31,39,40,41]. Both genetic depletion of UCHL1 as well as transfection to introduce a catalytically inactive UCHL1 mutant reduced the ability of various cancer cell lines to metastasize [42,43]. This suggests that pharmacological inhibition of UCHL1 is a potential therapeutic avenue for treatment of UCHL1-implicated cancers.

**Figure 1:**
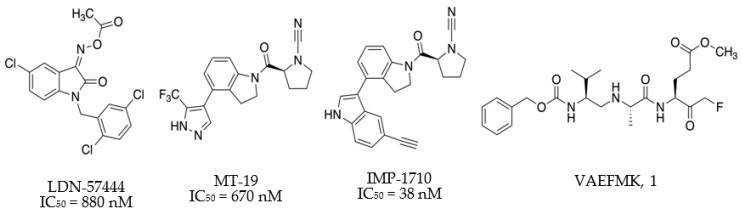
Previously reported UCHL1 inhibitors with biochemical IC_50_ values versus UCHL1.

The previous gold standard UCHL1 probe was the reversible inhibitor LDN-57444 [44] (Figure 1), however, the utility of this molecule as a UCHL1 inhibitor has been questioned recently as on-target engagement of UCHL1 in cellular environments has not been observed [45]. An alternative strategy for targeting UCHL1 is via electrophilic conjugation of the catalytic cysteine, Cys90. A recent set of reversible cyanamide-based covalent inhibitors have been described represented by MT-19 [46] and IMP-1710 [45] (Figure 1). MT-19 displayed a UCHL1 IC_50_ value of 670 nM after 30-min pre-incubation while IMP-1710 displayed a value of 38 nM. However, these inhibitors may suffer from apparent non-selective toxicity, particularly in non-UCHL1 expressing cells. Krabill et al. [46] showed MT-19 displayed anti-proliferative properties in KMS-12 cell lines that do not express UCHL1, and IMP-1710 began to display cytotoxicity at 10 µM in patient derived human bronchial fibroblasts [45]. 

The tripeptide benzyloxycarbonyl-Val-Ala-Glu(γ-methoxy)-fluoromethylketone (VAEFMK, **1**) (Figure 1) represents an alternative class of covalent UCHL1 inhibitor that has remained unexplored. VAEFMK was originally discovered serendipitously as a hit in a screen of halo-methylketone tripeptides against the herpes simplex virus cysteine protease UL36 [47]. However, during counter-screening against a panel of human DUBs, VAEFMK was shown to inhibit UCHL1. The molecule was co-crystallized with UCHL1 by Davies et al., which remains the only ligand-bound UCHL1 complex reported to date [48]. However, there is little biochemical and cellular information reported for this scaffold. Covalent peptides have long been utilized as inhibitors for cysteine proteases including the known caspase inhibitor VADFMK [49]. The fluoromethylketone moiety is less reactive than chloromethylketone and cyanamide counterparts and is an irreversible inhibitor, as opposed to the reversibility of the cyanamides. Thus, we hypothesized VAEFMK would exhibit little off-target non-specific reactivity in cells, making it a suitable starting point for structure-activity relationship (SAR) studies. To this end, our group set out to fully characterize VAEFMK as a UCHL1 inhibitor in biochemical and cellular assays as well as carry out SAR studies on the scaffold to improve potency and understand the mechanism of action. Successfully characterizing this inhibitor and confirming on-target engagement in a cellular environment would serve to validate this molecular scaffold as a novel probe for UCHL1. The results of these studies are presented herein.

## 2. Results and Discussion

### 2.1. Synthesis of VAEFMK and Derivatives

**Scheme 1:**
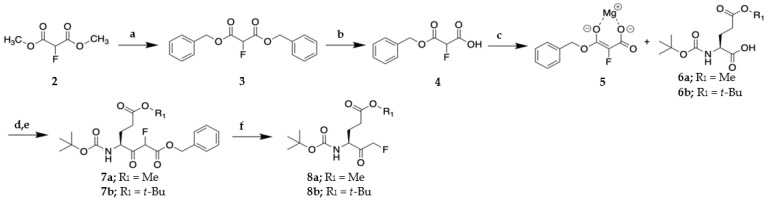
Synthesis of fluoromethylketone amino acid intermediates. Reagents and conditions: (**a**) benzyl alcohol (10 eq), TsOH (0.1 eq), toluene, 75 °C, 2 h; (**b**) NaOH (1.05 eq), *i*-PrOH, 45 °C, 70 min; (**c**) isopropyl MgCl (2.0 eq), THF, 0 °C, 1 h, crude to next step; (**d**) **6** (1.0 eq), CDI (1.1 eq), THF, 0 °C, 1 h, crude to next step; (**e**) **5** (2.0 eq), THF, −20 °C—RT, 3.5 h, crude to next step; (**f**) H_2_, Pd/C, toluene, RT, overnight.

VAEFMK **1** and analogs were synthesized using variations to previously published protocols [50,51,52,53]. In brief, to prepare the FMK containing intermediates (Scheme 1) the key fluorinating reagent was synthesized via transesterification of dimethyl fluoromalonate **2** with benzyl alcohol, resulting in dibenzyl fluoromalonate **3**. Hydrolysis of a single benzyl group yielded key reagent 3-(benzyloxy)-2-fluoro-3-oxopropanoic acid (MBF) **4**. The reagents could be converted to the magnesium enolate **5** and added to an activated ester of either Boc-Glu(OMe)-OH (**6a**) or Boc-Glu(OtBu)-OH (**6b**), forming intermediates **7a**-**b**. These intermediates were isolated and immediately hydrogenated using H_2_ and Pd/C to yield the key building blocks Boc-Glu(OMe)-FMK **8a** or Boc- Glu(OtBu)-FMK **8b**.

**Scheme 2:**
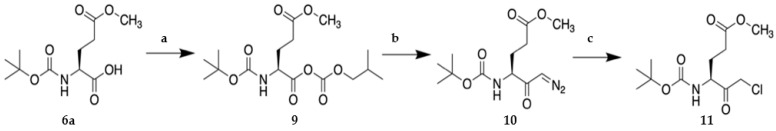
Synthesis of Boc-Glu(OMe)-CMK. Reagents and conditions: (**a**) *N*-methylmorpholine (1.3 eq), isobutyl chloroformate (1.2 eq), THF, 0 °C, 45 min; (**b**) Diazald^®^ (2.2 eq), carbitol (1 mL), ether (1 mL) 37% *w*/*v* KOH, 0 °C, 2 hr; (**c**) 4 M HCl in 1,4-dioxane, until solution is not yellow, 0 °C, 30 min.

The corresponding chloromethylketone (CMK) intermediate was prepared according to Scheme 2 from **6a**. The carboxylic acid was activated by isobutyl chloroformate to form the mixed anhydride **9**, followed by the nucleophilic addition of diazomethane, generated in situ with Diazald^®^, to form a diazoketone **10**. Quenching the reaction with HCl in 1,4-dioxane provided the building block Boc-Glu(OMe)-CMK **11**.

**Scheme 3:**
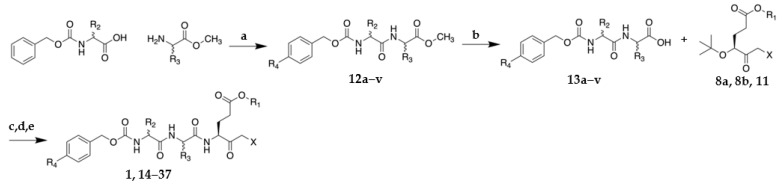
Synthesis of Cbz-protected tripeptide halomethylketones. Reagents and conditions: (**a**) Cbz-carboxylic acid (1.0 eq), methyl ester amino acid (1.0 eq), *N*-methylmorpholine (4.0 eq), isobutyl chloroformate (1.5 eq), THF, 0 °C–RT, overnight; (**b**) LiOH (1.2 eq), 4:1 THF:H_2_O, 0 °C, 3 h. (**c**) **8a**, **8b**, or **11** (1.0 eq), 4.0 M HCl in 1,4-dioxane (7.4 eq), 30 min, RT, carried into d; (**d**) i. carbamate-protected dipeptide acid **12a**–**s** (0.8 eq), *N*-methylmorpholine (4.0 eq), isobutyl chloroformate (1.5 eq), THF, 0 °C, 45 min; ii. addition of Boc-deprotected **8a**, **8b**, or **11**, THF, 0 °C–RT, overnight; (**e**) For analogs that require side-chain deprotection—precursor tripeptide intermediate (1.0 eq), 95:2.5:2.5 *v*:*v*:*v* TFA:TIPS:H_2_O, 1 h, room temperature.

These building blocks were then combined into the synthesis of the final benzyloxy (Cbz)-protected tripeptide analogs according to Scheme 3. Diversity at R_1_ and R_2_ amino acid side chains were introduced at this point of the synthesis to generate the Cbz-protected dipeptide methyl ester intermediates **12a**–**t**. The ester was then cleaved to provide the free carboxylic acids **13a**–**s** that were carried into the final reaction crude. The analogous intermediates **12t**–**v** were then fed into the synthesis of final analogs as shown in Scheme 3. The Boc-protected halomethylketone intermediates **8a**, **8b**, and **11** were deprotected to provide the free amines, which were then carried into the final peptide coupling step and paired with **13a**–**v** to yield the final electrophilic analogs. Several analogs, including the Glu-fluoromethylketone derivative, required side-chain protecting groups to be removed and were subjected to deprotection to arrive at the final analog. 

For further investigation in cellular engagement assays four analogs were synthesized with modification to the benzyloxy-capped end of the tripeptide. The first set installed a *p*-ethynylbenzyloxy capping group to be leveraged for click chemistry. The synthesis of these analogs (Scheme S1) began by generating the *p*-ethynylbenzyl chloroformate from 4-ethynylbenzyl alcohol and triphosgene. This reagent was used to cap the corresponding dipeptide methyl esters to provide **12u**–**v**. These intermediates were fed into the synthetic route outlined by Scheme 3. to generate the carboxylic acid intermediates **13u**–**v**, which were then coupled with the corresponding Glu(OMe) halomethylketones to arrive at analogs **34**–**37**. 

### 2.2. Structure-Activity Relationship Studies for Tripeptide Halomethylketones

**Figure 2:**
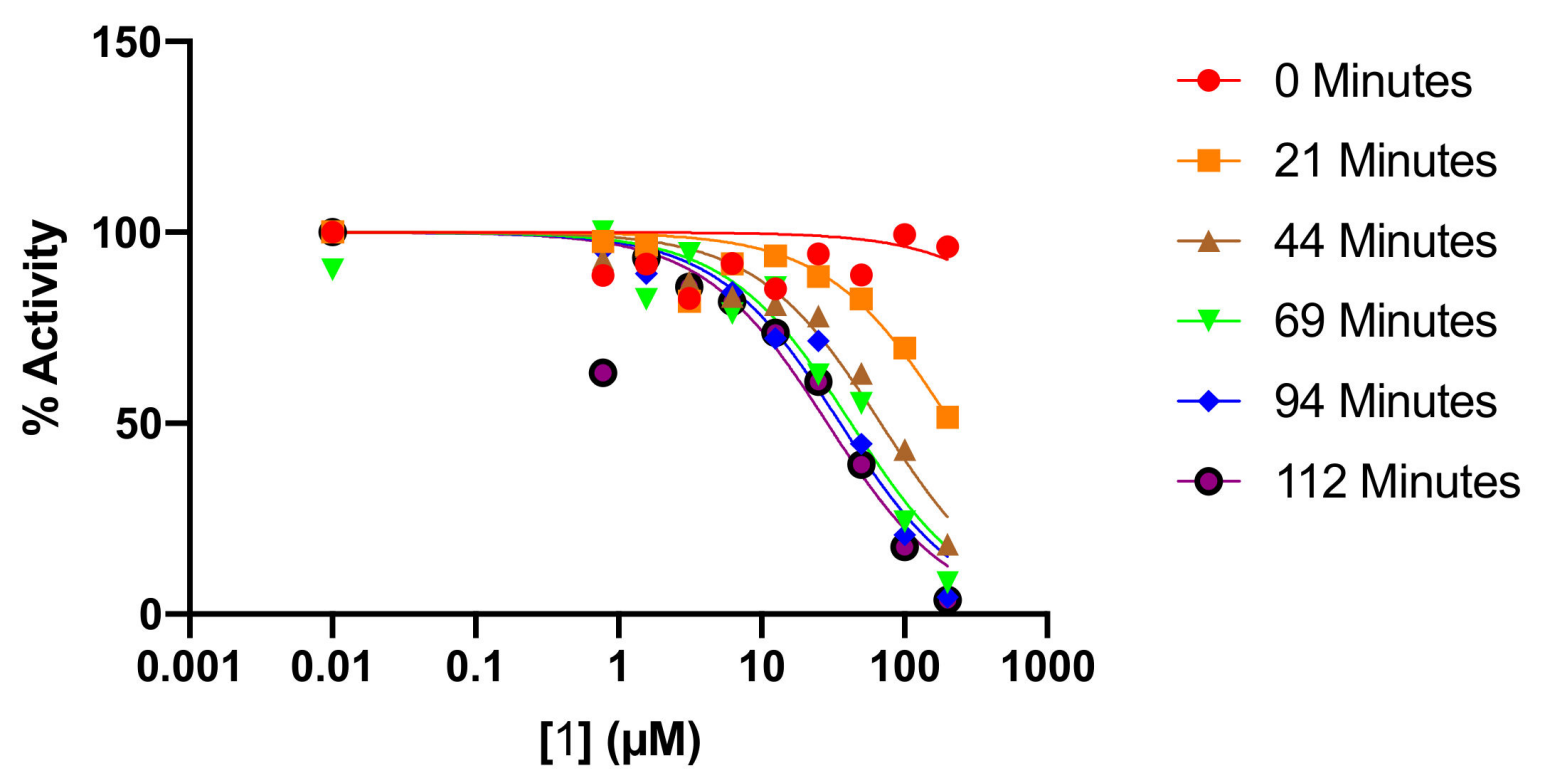
Time-dependent inhibition of UCHL1 by **1**. IC_50_ curves for His-UCHL1 treated with **1** at various points in time shows increasing potency with increasing length of incubation.

The in vitro inhibition of UCHL1 was determined by monitoring the cleavage of rhodamine 110 from substrate Rhodamine110-ubiquitin (Ub-Rho) [54]. As a putative irreversible inhibitor of UCHL1, the potency of **1** did increase with increasing length of preincubation, indicative of the irreversible nature of inhibition (Figure 2). After approximately 112 min, the molecule had reached near maximum efficacy, with the half maximal inhibitory concentration (IC_50_) value of 29 µM and 24 µM at 3-h incubation. Thus, a 3-h preincubation period was selected for the structure-activity relationship (SAR) comparison of all analogs to ensure full reaction time and for consistent comparison of activity against UCHL1 within the scaffold. The initial molecule **1** was reported to not possess any activity toward two closely related UCH family DUB, UCHL3 and UCHL5, at concentrations up to 400 µM [48]. This was confirmed in our assay with no activity versus UCHL3 (Figure S1) at the top concentration of 200 µM. Therefore, during SAR we did not assess off-target inhibition of UCHL3 or UCHL5, instead we chose to assess selectivity of our final compounds when the time came.

The in vitro efficacy of covalent inhibitors can be broken down into the reversible interactions that contribute to binding affinity and the covalent reactivity of the electrophilic “warhead” with the catalytic Cys. The fluoromethylketone of **1** is very slow to react with the active site cysteine, requiring approximately 2 h to reach nearly full inhibition. Chloromethylketones are more reactive than fluoromethyl ketones, and have been shown to be 3-fold more effective in matched molecular pair analogs [49]. Therefore, we reasoned that the efficacy of **1** could be improved simply by increasing the reactivity of the warhead. To test this hypothesis, the fluoromethylketone of **1** was substituted for a chloromethylketone, resulting in compound **14** (Table 1). Surprisingly, subsequent testing revealed a complete loss of in vitro activity against UCHL1 for the chloromethylketone analog. DTT is commonly used in this assay to ensure the active site cysteine remains in a reduced state to carry out its catalytic function. However, it can also act as a bio-nucleophile leading us to hypothesize that the excess DTT may be forming an adduct with the highly reactive chloromethylketone and effectively impairing the activity of the molecule. 

**Figure 3:**
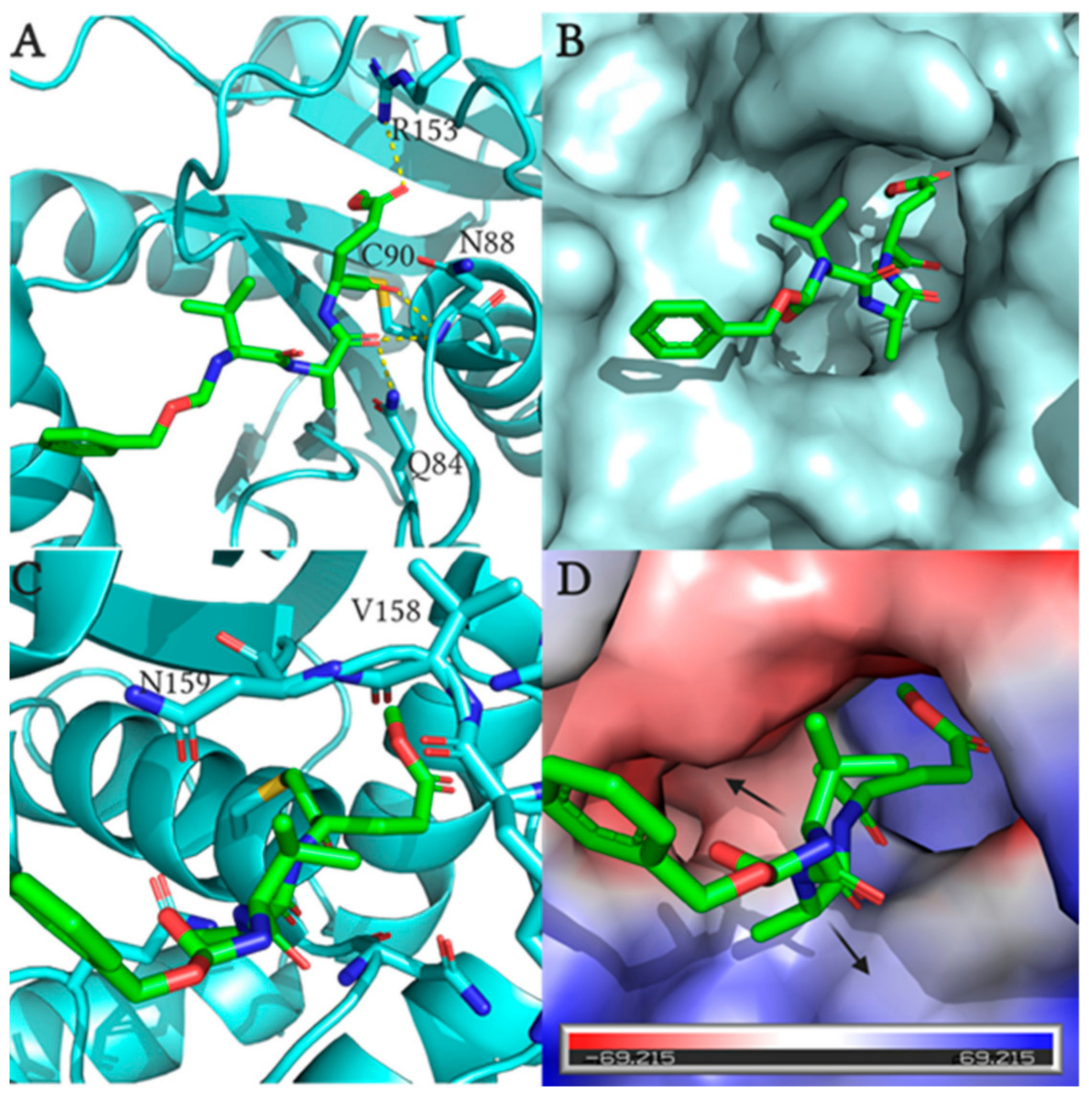
Crystal structure of VAEFMK **1** (green sticks) bound to UCHL1 (cyan). (**A**) VAEFMK contacts (yellow dashed line) with critical residues in UCHL1 active site. (**B**) Surface representation of VAEFMK binding pose in UCHL1 active site. (**C**) Point of view depicting that valine in VAEFMK is directed toward cross-over loop amino acid residues Val158 and Asn159. (**D**) Electrostatic surface map representation with VAEFMK bound (generated in PyMol). Red regions indicate negative surface charge and blue indicates positive surface charge. Arrows indicate the vector in which the *D*-amino acid side chains for valine and alanine would be directed from VAEFMK.

Analysis of the UCHL1-**1** co-crystal structure (Figure 3A) shows a hydrogen bond is observed between the carbonyl oxygen of the fluoromethylketone and the amide nitrogen of Cys90, as well as the ε^2^ nitrogen of the UCHL1 Q84 side chain [46]. The amide carbonyl oxygen between the glutamic acid methylester and alanine of **1** forms a hydrogen bond with backbone amide of Asn88. Finally, the ε^2^ oxygen of the glutamic acid methyl ester of **1** forms a hydrogen bond with an Arg153 side chain guanidine proton. Thus, we hypothesized that removal of the glutamic acid methylester from analog **15** to form the glutamate side chain (**16**) may provide an opportunity to form a salt-bridge with Arg153. Unexpectedly, this modification resulted in a complete abrogation of activity, even after 6 h of incubation with the enzyme. One possible explanation for the loss of efficacy is the potential for an intramolecular cyclization between the glutamate oxygen and the fluoromethylketone carbonyl, that could result in the formation of a six-membered lactone (Figure S2). A similar observation has been documented for aspartic acid fluoromethylketone, which undergoes a pH dependent cyclization as observed by NMR [50]. This would need to be further investigated to confirm the cyclization in future work.

SAR at R_2_ began with substitution of the enantiomer *D*-valine (**17**) in place of the natural *L*-valine found in **1** and this modification led to a complete loss of activity (Table 1). Additionally, removal of the valine side chain entirely (**18**) results in a complete abrogation of activity against UCHL1 where as an alanine at R_2_ (**19**) rescued activity, though the molecule was 3-fold less effective (IC_50_ = 76 µM) than parent compound **1**. Increasing the chain length by substitution of a leucine (**20**, IC_50_ = 23 µM) or phenylalanine (**21**, IC_50_ = 13 µM) led to improvement over the parent molecule **1**. The trend observed at R_2_ to be Phe > Leu > Val > Ala > Gly clearly indicates the preference for a bulky, hydrophobic residue at this position and the need for the natural amino acid stereochemistry. Further inspection of the VAEFMK-bound structure of UCHL1 (Figure 3C) indicates that the valine is directed toward the flexible crossover loop of UCHL1 [55]. This would explain why bulkier hydrophobic residues were preferred at R_2_ as there is space to accept such a modification. There is little room for any *D*-enantiomeric substituent at R_2_ as the vector (Figure 3D, top arrow) representing the *D*-valine substitution in analog **17** would be directed toward and clash with the surface of UCHL1 explaining the lack of activity for this analog. 

Insertion of polar side chains reduced UCHL1 activity. For example, the serine containing derivative at R_2_ displayed much reduced activity with an IC_50_ = 185 µM. However, increasing the lipophilicity of this polar side chain by incorporating a threonine, which more resembles the steric size of the parent valine side chain, displayed an IC_50_ value of 100 µM demonstrating that the lipophilic bulk can mitigate some of the loss of activity due to incorporation of the polar hydroxyl group. Moving to an asparagine, which is similar in size to a leucine but contains the polar amide side chain, maintained activity comparable to the threonine with an IC_50_ value of 119 µM, however, when this position was modified with an acidic aspartate side chain, which would be charged at physiological pH, the molecule completely lost activity even at the highest concentrations tested. The observations from this subset of analogs indicate that non-ionizable polar side chains but do still exhibit some anti-UCHL1 activity but are not preferred, and that this activity can be improved with addition of hydrophobic atoms. However, introduction of an ionizable side chain abrogated activity of the scaffold. The electrostatic potential surface representation (Figure 3D) shows that the surface that the side chains would be directed toward carries a partial negative charge, possibly explaining the aversion to residues that carry a negative ionizable charge as this may result in electrostatic repulsion from the binding site.

The same modifications were attempted at R_3_ as were built in at R_2_ with limited success. Similar to the R_2_ position, the substitution of the *D*-alanine (**26**) in place of the parent *L*-alanine resulted in loss of activity. Only the glycine substituent (**15**) retained any activity, albeit much reduced (IC_50_ = 95 µM) compared to the parent **1**, while the remaining analogs all exhibited IC_50_ values > 200 µM. Again, inspection of the ligand bound structure explains the observed results as alanine at R_3_ is directed toward the UCHL1 surface in a tight space that would not be amenable to increased steric bulk (Figure 3D). This is evidenced by the fact that the glycine analog retains some activity, but also indicates the methyl group present in the alanine derivative does provide some level of hydrophobic binding. Finally, the direction in which the *D*-alanine would be projected toward also would clash with UCHL1 surface offering an explanation for the loss of efficacy with this analog.

In preparation for cellular target engagement studies the benzyloxy capping group was modified to install a *para*-alkyne handle amenable to click chemistry at R_4_. Interestingly, each derivative that had the 4-alkynylbenzyloxy installed was actually more potent than the nearest neighbor benzyloxy analog. For example, the 4-alkyne containing analog **34** displayed an IC_50_ value of 7.7 µM compared to the matched molecular pair **1** for an increase of approximately 3-fold activity versus UCHL1. The same trend was observed for **35**, which contained a glycine at R_3_ and the 4-alkyne at R_4_, being about 1.5-fold more active than the non-alkyne containing analog **15**. The trend was not as noticeable when comparing **36**, with a phenylalanine at R_2_ and alkyne at R_4_, to the non-alkyne derivative **21**, although there was slight improvement observed. As a means of comparison, an alkyne containing derivative of the chloromethylketone (**37**) was also produced but this molecule was still inactive in the in vitro assay. The improvement of activity by installation of the alkyne was somewhat surprising as the benzyloxy group in the crystal structure was found to be solvent-exposed and not taking part in any specific interactions; nonetheless, the data suggests that there may be opportunity to further explore SAR on the phenyl ring in future work.

**Figure 4:**
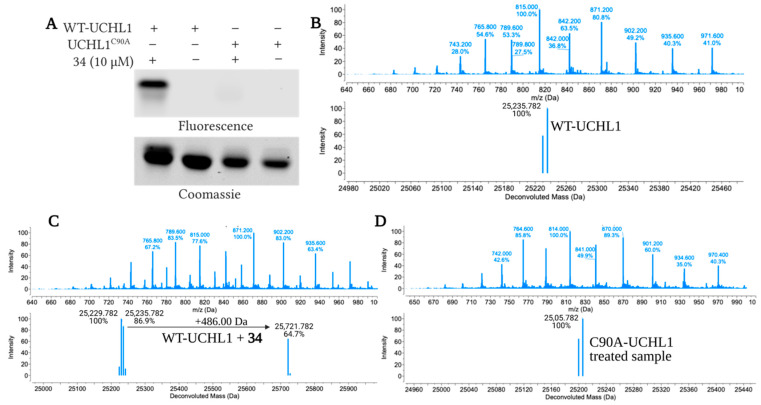
Fluoromethylketone **34** selectively conjugates to the Cys90. (**A**) Recombinant WT-UCHL1 and catalytically inactive C90A mutant followed click reaction with Cy5-Azide and analyzed by in-gel fluorescence shows the alkyne-tagged analog **34** forms a covalent adduct with WT-UCHL1 but does not form an adduct with the catalytically inactive C90A mutant. Coomassie stain provided as loading control. (**B**) Deconvoluted mass spectrum for WT-UCHL1 at 10 µM. (**C**) Deconvoluted mass spectrum for WT-UCHL1 (10 µM) treated with **34** (20 µM). (**D**) Deconvoluted mass spectrum for C90A-UCHL1 (10 µM) treated with **34** (20 µM).

In addition to the catalytic Cys90 residue, UCHL1 possesses five other cysteines. Among the five alternative cysteine residues Cys152 has previously been reported to exhibit nucleophilic activity toward endogenous electrophiles [56,57] as well as be involved in trans-nitrosylation events [58], while Cys220 has been reported to play a role in farnesylation [59] and AKT signaling [35]. A common shortcoming to covalent inhibitors is that the reactive electrophilic groups may partake in non-specific labeling of off-target cysteines. To investigate if alternative cysteines are affected on UCHL1 we used analog **34** to confirm that the fluoromethylketone derivatives only conjugate Cys90. Molecule **34** was incubated with both recombinant wild-type UCHL1 containing Cys90 and catalytically inactive UCHL1 that possesses a C90A mutation, then a click reaction was performed to append a fluorophore for in-gel fluorescence imaging. It was observed that analog **34** only formed a covalent adduct with the Cys90 of the WT-UCHL1 while no fluorescent bands were observed for the UCHL1^C90A^ treated protein (Figure 4). Additionally, samples were analyzed by mass spectrometry to increase sensitivity for detection of any non-Cys90 adducts. The mass spectrometry data unequivocally suggests that **34** reacts only with Cys90 of UCHL1 as a single adduct + 486.00 Da was observed that would correspond to **34** without the fluorine atom (Figure 4C). There were no adducts observed in the UCHL1 C90A samples incubated with **34** (Figure 4D). Table 2 summarizes the mass spectrometry data from the samples tested. This data confirms that the fluoromethylketone electrophile is sufficiently selective to the catalytic Cys90 on UCHL1.

To summarize thus far, our SAR studies revealed that *L*-amino acids were superior to *D*-amino acids at R_2_ and R_3_. Bulky, lipophilic side chains were preferred for maintaining or improving activity against UCHL1 at R_2_, whereas any increase of steric bulk at R_3_ was detrimental to activity. Polar functional groups reduced activity at both locations. Finally, the derivatives with alkynes at R_4_ all outperformed the corresponding 4-H analogs to varying degrees, with molecule **34** being the most potent in terms of the single time-point efficacy versus UCHL1 with an IC_50_ value of 7.7 µM, lending evidence that there may be additional room for improvement in modifying the benzyloxy cap to the peptide. This molecule also maintained no activity against UCHL3 at > 200 µM (Figure S1), confirming the superior selectivity over the closest related DUB family member. It should be noted that while no modifications were made to alter the Glu side chain beyond de-methylation of the ester, previous studies have confirmed that the well-known pan caspase inhibitor VADFMK [60], which differs from VAEFMK by an Asp(OMe) in place of the Glu(OMe), is also inactive against UCHL1 [48]. Taken together, it is apparent that the Glu(OMe) is crucial to position the electrophile in just the right orientation to undergo the covalent reaction in the context of the relatively inert fluoromethylketone electrophile and the active site Cys90 of UCHL1. 

### 2.3. Kinetic Analysis of Fluoromethylketone Analogs

**Figure 5:**
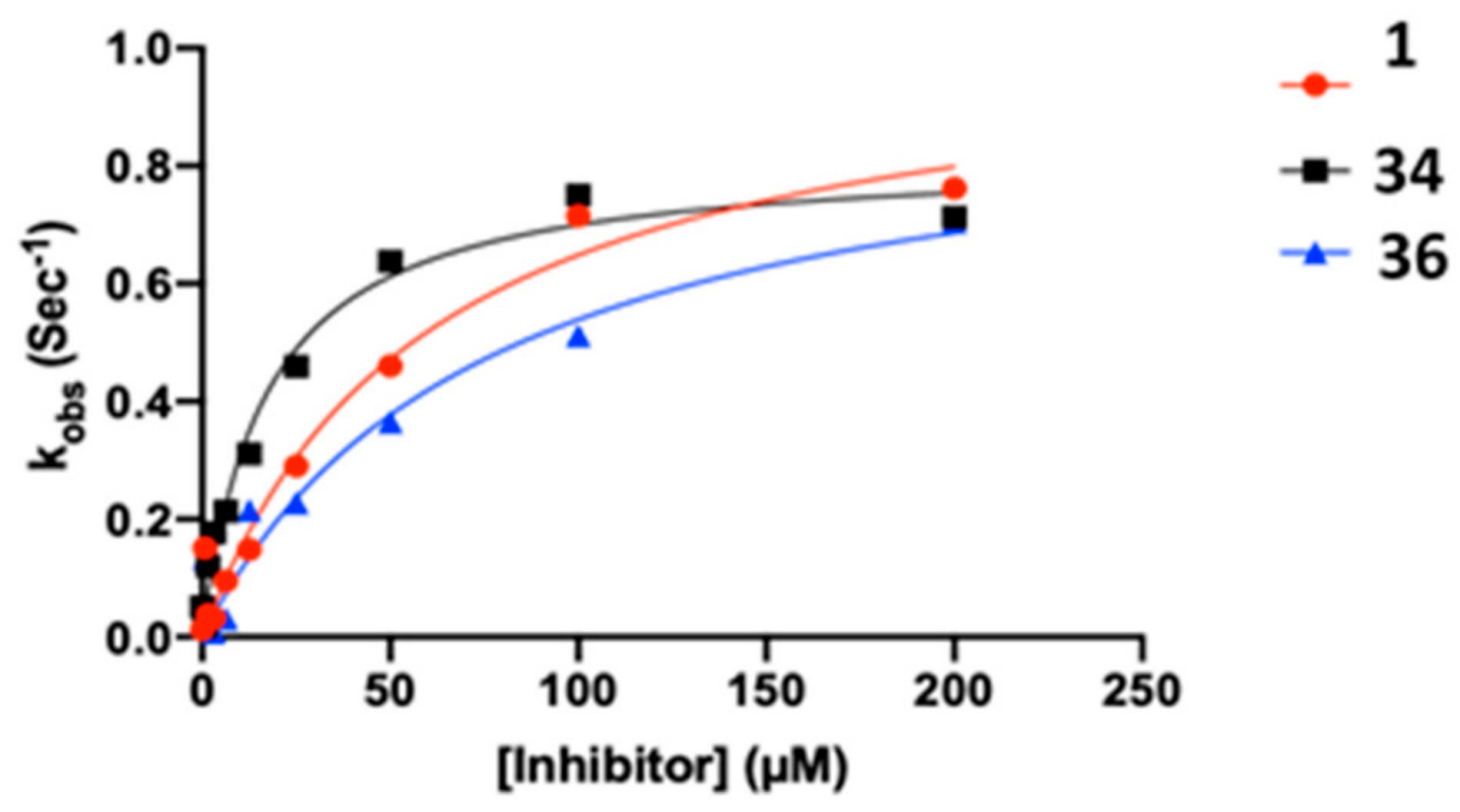
Inactivation kinetics for FMK analogs. *k*_inact_/*K*_I_ plots for UCHL1 treated with **1**, **34**, and **36** (Values are 288.05 M^−1^S^−1^, 813.3 M^−1^S^−1^, and 209.9 M^−1^S^−1^ for **1**, **34**, and **36**, respectively.

Irreversible covalent inhibitors may be further characterized by determining the reversible binding constant (*K*_I_) as well as the rate of the covalent bond formation under saturating conditions (*k*_inact_) [61,62,63]. By determining the IC_50_ over the course of various timepoints, the pseudo first-order rate constant (*k*_obs_) could be calculated. Plotting *k*_obs_ as a function of inhibitor concentration generated a curve that could be fit using the equation Y = *k*_inact_*X/(*K*_I_ + X) (Figure 5) [64]. For parent compound **1**, these values were determined to be *K*_I_ = 60.04 µM and *k*_inact_ = 0.0173 s^−1^, resulting in a *k*_inact_/*K*_I_ value of 288.05 M^−1^s^−1^ (Table 3). In comparison, the *k*_inact_/*K*_I_ values for the two more potent analogs from the SAR studies were 810.0 M^−1^s^−1^ and 208.4 M^−1^s^−1^ for **34** and **36**, respectively. The improvement in both IC_50_ and *k*_inact_/*K*_I_ for **34** appears to be attributed to an increase the affinity of the analog towards UCHL1 as the *K*_I_ was improved greater than 3-fold. The fact that **36** was essentially equipotent to **1** in the covalent kinetics was somewhat unexpected as the previous single time-point assays with a set preincubation of 3-h showed the molecule to be 2-fold more potent than **1**. However, this could likely be due to a difference in the assay conditions as far as preincubation times because covalent inhibitors become more potent over time [63]. This underscores the importance of determining the *k*_inact_/*K*_I_ value for key analogs to robustly determine inhibitor efficacy and further confirmed the superiority of **34** compared to the original parent molecule.

### 2.4. Fluoromethylketone Analogs Preclude Binding of Ub to UCHL1

**Figure 6:**
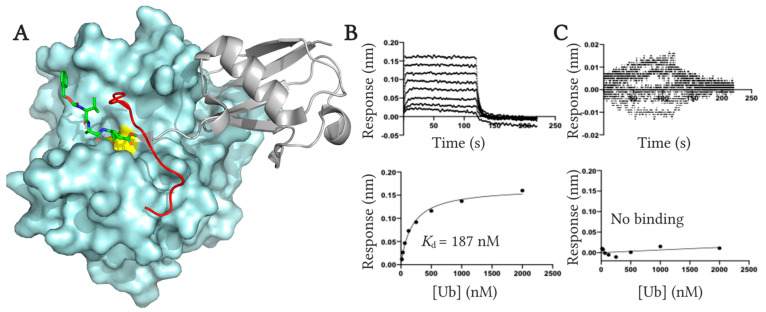
Competition of binding for **1** versus Ub. (**A**) Overlaid crystal structure of **1** (green sticks) bound to UCHL1 (cyan surface) (PDB: 4DM9) and Ub (gray ribbon) bound to UCHL1 (PDB: 3KW5). Crossover loop of UCHL1 shown in red ribbon for clarity. (**B**) Association/dissociation and steady-state binding data for UCHL1 and 1:1 serial dilutions Ub in BLI assay buffer. (**C**) Association/dissociation and steady-state binding data for UCHL1 after preincubation with **1**.

In addition to its role as a deubiquitinating enzyme other putative physiological functions for UCHL1 under normal condition are hypothesized to be to (1) maintain a mono-Ub pool within cells [65,66] and (2) mask sites of activation on Ub through maintaining the Ub-UCHL1 protein-protein interaction (PPI) [67,68]. Given this, and the relatively strong binding affinity of Ub to UCHL1, it is believed that within cells UCHL1 is mostly bound to free mono-Ub. The ligand-bound structure of **1** bound to UCHL1 confirms that the inhibitor does not bind to the Ub-binding site of UCHL1, but actually approaches and binds to the face opposite of the Ub-site and interacts with the catalytic cysteine (Figure 6A, PDB: 4DM9 [48] and PDB: 3KW5 [55]). This would seemingly still allow the Ub-binding interface to be available to interact with mono-Ub. To determine if **1** is able to abrogate the UCHL1:Ub interaction, Ub-binding studies were carried out using biolayer interferometry (BLI). His-UCHL1 was preincubated with an excess of **1** (2 mM) or DMSO overnight at room temperature to ensure full covalent modification of the Cys90 was achieved. The dissociation constant (*K*_d_) of Ub towards UCHL1 pre-treated with DMSO treated control was determined and proved to be similar to previously published affinities (Figure 6B) [69]. However, the presence of **1** conjugated to the Cys90 completely abrogated the ability of UCHL1 to interact with Ub (Figure 6C). Taken together, these data suggest that even though **1** binds on the opposite face of UCHL1 compared to the Ub-binding domain it still completely precludes Ub binding. This may be a factor of **1** stabilizing the crossover loop and, in turn, precluding Ub binding; however, further investigation is needed to test this hypothesis. Nonetheless, it appears the inhibition of UCHL1 by the fluoromethylketone analogs would reduce the binding of mono-Ub within the cell to UCHL1.

### 2.5. Cellular Efficacy in Cancer Cell Lines

Levels of UCHL1 expression are correlated with increased metastatic behavior and poor patient prognosis in a number of cancers. To evaluate 1 and analogs in a cellular context, three previously validated cell lines were utilized based on their sensitivity to UCHL1 depletion: SW1271 (small-cell lung cancer), KMS11 and KMS12 (myeloma cell lines). 

#### 2.5.1. VAEFMK Efficacy in Myeloma Cells

**Figure 7:**
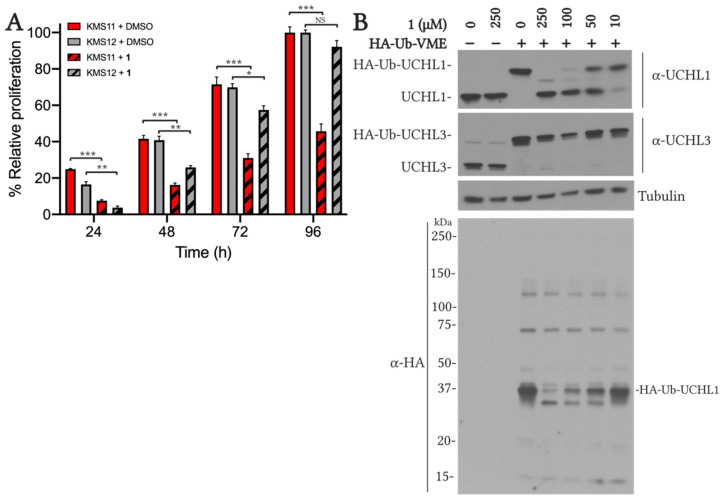
Cellular characterization of **1** in KMS11 and KMS12 myeloma cells. (**A**) Cellular proliferation data for KMS11 and KMS12 cells treated with **1** (100 µM) or DMSO control at 24-, 48-, 72-, and 96-h timepoints. N = 3, *, *p* < 0.05; **, *p* < 0.01; ***, *p* < 0.001. Statistical significance calculated using student’s *t*-test (**B**) Immunoblots data for KMS11 cells treated with **1** (0–250 µM) then reacted with HA-Ub-VME. Blotting for UCHL1 (top panel) indicates the molecular weight shift of the HA-Ub-UCHL1 complex in the control lane and the dose-dependent decrease of this complex formation in KMS11 cells. Blotting for UCHL3 (second panel from top) indicates formation of HA-Ub-UCHL3 complex at every dose. Blotting for HA (bottom panel) confirms only dose-dependent decrease of HA-Ub-UCHL1 complex formation and no dose-dependent decrease of any other DUB.

KMS11 cells endogenously express UCHL1 and are sensitive to genetic depletion of UCHL1 using shRNA, whereas KMS12 cells do not express UCHL1 and are not sensitive to genetic depletion These cells provide a good control for evaluating off-target effects of UCHL1 inhibitors [36,46]. Consistent with previously reported sensitivity to UCHL1 depletion [34], KMS11 cells dosed with 100 µM **1** exhibited reduced proliferation compared to DMSO controls after 24, 48, 72 and 96 h timepoints (Figure 7A). Alternatively, KMS12 cells, which are not sensitive to UCHL1 genetic depletion, showed reduced growth at the 24-h timepoint but regained proliferative ability at the 96-h timepoint. The KMS12 cells are less sensitive to **1** than KMS11 and the molecule clearly displays a differential effect on KMS11 and KMS12 cells that would indicate they selectively inhibit UCHL1 in KMS11 cells. 

To confirm on-target engagement in KMS11 cells, a Ub activity-based probe (ABP) gel-shift assay was employed [70]. Using the hemagglutinin-tagged Ub-vinylmethylester (HA-Ub-VME) ABP to examine the activity of **1** in KMS11 cells, an immunoblot was performed. Cells were treated in dose-response with **1** (0–250 µM), washed with PBS, then lysed and treated with HA-Ub-VME. The HA-Ub-VME labels any remaining DUBs that have not been inhibited by **1**. Blotting for UCHL1 showed a dose-dependent decrease in HA-Ub-UCHL1 complex formation with increasing concentration of **1**, confirming on-target engagement of UCHL1 in cells (Figure 7B, top panel). The same blot was performed for UCHL3 to confirm the molecule does not inhibit the closely related DUB in cells. The blot shows full formation of the HA-Ub-UCHL3 complex at all concentrations after preincubation with **1** indicating the molecule does not inhibit UCHL3 in cells (Figure 7B, second panel). HA-Ub-VME reacts with all DUBs in lysates; thus, blotting for HA qualitatively reveals the broader DUB selectivity of the inhibitor. This blot revealed that **1** exhibited the expected dose-dependent decrease in HA-Ub-UCHL1 complex formation (Figure 7B, bottom panel). However, a higher exposure of the blot did show another DUB corresponding to the molecular weight UCHL5 may also be inhibited at 250 µM (Figure S4). No other DUBs in the KMS11 cell line revealed any dose-dependent changes in complex formation upon treatment with **1** suggesting selectivity for UCHL1 within the DUBs expressed in this cell line and corroborating the observed UCHL1-dependent reduction in cell proliferation. 

#### 2.5.2. Cell Viability and On-target Engagement of Analogs in SW1271 Small Cell Lung Cancer Cells

**Figure 8:**
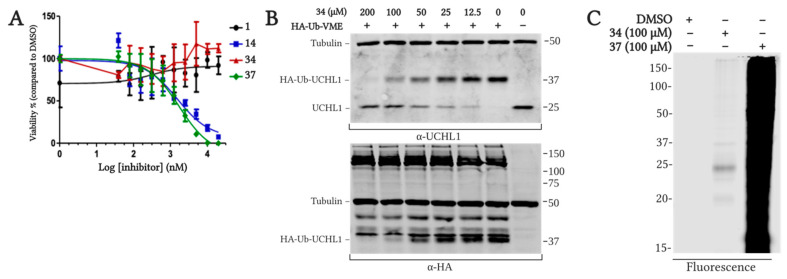
SW1271 small cell lung cancer cell viability and on target engagement of inhibitors. (**A**) Cell viability of the SW1271 cell line in the presence of selected fluoromethylketone and chloromethylketone analogs. (**B**) Ub-ABP assay showing on-target engagement of fluoromethylketone **34** with UCHL1 in a dose-dependent manner (top panel). HA immunoblot shows selectivity for UCHL1 over other DUBs within SW1271 cells (bottom panel). Blotting for tubulin loading control shown on same HA-blot (**C**) In-gel fluorescence of SW1271 cells treated with fluoromethyl ketone **34** and chloromethylketone **37** then subjected to click reaction with Cy5-azide fluorophore.

UCHL1 is a key promoter of cell invasion and metastasis in small cell lung cancer but not necessarily required for cell viability or proliferation [43,71]. Thus, we chose to assess the efficacy of targeting UCHL1 using the SW1271 small cell lung cancer cell line. Treatment of SW1271 cells with fluoromethylketones **1** and **34** (100 µM) has no effect on the cell viability, whereas chloromethylketones **14** and **37** both kill the cells in a dose-dependent manner with CC_50_ values of 0.156 µM and 0.176 µM, respectively (Figure 8A). The Ub-ABP assay was used to confirm on target engagement of UCHL1 within these cells. Analog **34** inhibited HA-Ub-VME complex formation with UCHL1 in a dose-dependent manner (Figure 8B, top panel) and demonstrated selectivity over other DUBs in SW1271 cells as the only band in the HA-blot to exhibit significant dose-dependent signal reduction was the Ub-UCHL1 band (Figure 8B, bottom panel).

Leveraging the alkyne handle, a click reaction was performed with an azide containing sulfo-cyanine5 fluorophore (Cy5) to visualize proteins within the cells that were conjugated to either the fluoromethylketone **34** or the chloromethylketone **37**. Cells were pretreated with either analog then harvested and washed to removed unreacted inhibitor. The cells were then lysed and the click reaction was performed on lysates. The fluoromethylketone displayed high selectivity in cells as the only dark band observed was that of a molecular weight corresponding to UCHL1 (approx. 25 kDa) with very light bands at other molecular weights that are likely a result of weak non-specific activity at other cysteines (Figure 8C). Conversely, the chloromethylketone **37** was observed to non-specifically label multiple proteins within the cell (Figure 8C). The degree of promiscuity observed for the more reactive chloromethylketone warhead likely explains the cytotoxicity against the SW1271 cell line, while the less reactive and more selective fluoromethylketone **34** did not reduce SW1271 cell viability. Further evaluation using proteomic profiling is warranted to gain a quantitative understanding on the potential for non-specificity at off-target cysteines.

#### 2.5.3. Inhibition of UCHL1 Reduces Migratory Capability of SW1271 Cells

**Figure 9:**
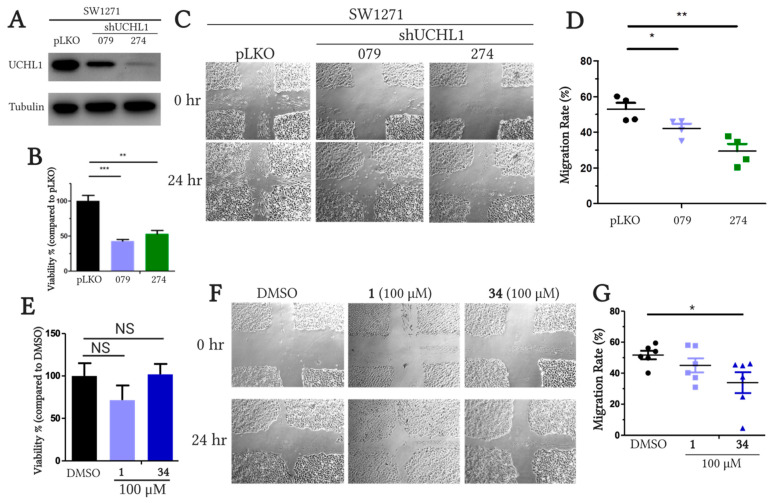
Effect of UCHL1 on migration of SW1271 cells. (**A**) Knockdown of UCHL1 in SW1271 cells. Shown are vector control (pLKO) and shRNA 079 and 274. (**B**) Effect of shRNA knockdown on SW1271 cell viability compared to pLKO control. (**C**) Wound healing cell images on SW1271 pLKO and UCHL1 knockdown cells at 0- and 24-h time points. Representative replicates shown for each. (**D**) Migration rate in % area covered by cells in 24-h period compared to pLKO control. (**E**) Effect of compounds **1** and **34** on cell viability at 100 µM dose compared to vehicle control (DMSO). (**F**) Wound healing cell images of wild type SW1271 cells treated with fluoromethylketone inhibitors at 0 and 24-h time points. Representative replicates shown. (**G**) Migration rate in % area covered by cells in 24-h period after treatment with inhibitor. N = 6, * *p* < 0.05; **, *p* < 0.01, *** *p* < 0.001. NS = not significant. Statistical significance calculated using two-tailed *t*-test.

UCHL1 has been reported to regulate and contribute to cell invasiveness and metastatic behavior in small cell lung cancer. To assess the role of UCHL1 in cell migration, a wound healing assay was performed using control and UCHL1 depleted SW1721 cells. First, shRNA knockdown of UCHL1 in SW1271 cells was performed and efficient knockdown was observed with shRNA 079 and 274 compared to the vector control pLKO (Figure 9A, vector and shRNA information in Table S3). While these cells did exhibit reduction of viability (Figure 9B) the knockdown cell lines were stable and used in the wound healing assay. Genetic depletion of UCHL1 reduced the ability of SW1271 cells to migrate by approximately 20% compared to the pLKO control vector cells at 24 hrs (Figure 9C,D).

The same experiments were performed on wild type SW1271 cells treated with fluoromethylketone analogs. Molecules **1** and **34** were first assessed for their effect on cell viability at 100 µM to determine if any observed reduction in cell viability may influence the migration assays (Figure 9E). Contrary to how the cells behaved in the shRNA knockdown experiments, when dosed with molecules neither **1** nor **34** displayed cytotoxic effect on the SW1271 cells at these concentrations. However, consistent with shRNA results treatment of SW1271 cells with 100 µM of **34** also significantly reduced the ability of the cells to migrate compared to the DMSO control while the same reduction of migratory ability was not observed for the less potent **1** (Figure 9F,G). These results illustrate the utility of pharmacological inhibition using **34** compared to shRNA knockdown as the using the probe allows one to separate the impact of cell viability from the antimigratory activity of UCHL1. Due to the slow reactive nature of the inhibitor and the modest potency the anti-migratory efficacy of these analogs may be improved with longer duration of treatment or increasing the frequency of treatment. Further studies are underway to elucidate the role UCHL1 plays in the migratory cellular pathways using both genetic depletion and inhibitor approaches. Nonetheless, these data indicate that selective pharmacologic inhibition of UCHL1 reduced the invasive capabilities of SW1271 small cell lung cancer cells.

## 3. Materials and Methods

### 3.1. Chemistry

#### 3.1.1. General Experimental

^1^H-, ^13^C- and ^19^F-NMR spectra were recorded on DRX500 or ARX-800 spectrometers (Bruker, Billerica, MA, USA) in [D_6_] DMSO or CDCl_3_ with or without the internal standard of TMS at 0.05 or 0.1% *v*/*v*. The purity of all final compounds was > 95% purity as assessed by HPLC. Final compounds were analyzed on a 1200 series chromatograph (Agilent, Santa Clara, CA, USA). The chromatographic methods used were either: (A) Hypersil GOLD C18 column (Thermo Scientific, Waltham, MA, USA) 3 µM particle size, 150 mm length, 4.6 mm ID) or (B) a Thermo Scientific Hypersil GOLD C18 column (3 µM particle size, 250 mm length, 4.6 mm ID). UV detection wavelength = 254 nm; flow rate = 1.0 mL min^−1^; solvent = acetonitrile/water. Both organic and aqueous mobile phase contain 0.1% *v*/*v* trifluoroacetic acid. The mass spectrometer used is a CMS-L Compact Mass Spectrometer (Advion, Ithaca, NY, USA) with an ESI or an APCI ionization source. Samples are submitted for analysis using either the atmospheric solids analysis probe (ASAP) or flow injection analysis (FIA). Compounds were prepared according to the following protocols and are detailed below. Intermediates **13a** (Catalog #V4659, AK Scientific, Union City, CA, USA) and **13b** (Catalog #7975AH, AK Scientific) were purchased from commercial vendors. These intermediates were left in Scheme 3 for continuity.

#### 3.1.2. General Procedure for synthesis of tripeptide halomethylketones

Synthesis for analog **21** presented. All other analogs and alternative synthetic procedures reported in Supplementary Materials.

##### Dibenzyl 2-fluoromalonate (**3**)

A mixture of dimethyl 2-fluoromalonate **2** (2.0 g, 13 mmol, 1.0 eq), benzyl alcohol (7.0 g, 65 mmol, 4.9 eq,), p-toluenesulfonic acid monohydrate (150 mg, 0.80 mmol, 0.06 eq) in toluene (5.6 mL) were placed in a round bottom flask outfitted with a Dean-Stark apparatus and was heated with stirring to 75 °C in vacuo (27 mm of Hg) until all of the toluene had distilled. Then (75 mm Hg, 112 °C) for an additional 5 h. The mixture was cooled to 75 °C and isopropanol (15 mL) was added followed by hexanes (30 mL). The mixture was placed in the freezer and allowed to crystalize overnight. The product was filtered, washed with hexanes (2 × 30 mL), and dried overnight *in vacuo*, yielding **3** as a white solid (3.0 g, 9.9 mmol, 76%). ^1^H NMR (500 MHz, Chloroform-*d*) δ 7.64–7.28 (m, 10H), 5.37 (d, *J* = 48.0 Hz, 1H), 5.25 (s, 4H); APCI-MS: *m/z* 301.1 [M − H]^−^.

##### 3-(Benzyloxy)-2-fluoro-3-oxopropanoic acid (4)

Intermediate **3** (2.0 g, 6.6 mmol, 1.0 eq) was suspended in isopropanol (12 mL) and heated to 45 °C. 1.0 M aqueous NaOH (6.9 mL, 6.9 mmol, 1.1 eq) was added dropwise over 1 h. After an additional 10 min, the solution was concentrated to 5 mL and water (2.5 mL) was added. The pH was adjusted to 9 using saturated sodium bicarbonate and washed with DCM (5 × 5 mL) to remove benzyl alcohol. The pH of the solution was adjusted to 2.2 using 5 M HCl, and di-isopropyl ether (5 mL) was used to extract the product. The pH of the aqueous layer was adjusted to 1.9 using 5 M HCl, and further extracted with di-isopropyl ether (5 mL). The combined extracts were washed with brine (5 mL), dried over MgSO_4_, filtered, and concentrated in vacuo at 35 °C to provide an oily residue. This was triturated with hexanes (7 mL) overnight with stirring to give a solid. The solid was filtered and dried under vacuum to yield **4** as a white solid (0.42 g, 2.0 mmol, 30%). ^1^H NMR (500 MHz, Chloroform-*d*) δ 7.38 (d, *J* = 2.6 Hz, 5H), 5.40 (d, *J* = 47.9 Hz, 1H), 5.32 (d, *J* = 1.1 Hz, 2H).

##### Methyl (S)-4-((tert-butoxycarbonyl)amino)-6-fluoro-5-oxohexanoate (**8a**)

To vial #1 was added **4** (1.3 g, 6.3 mmol, 1.2 eq) and THF (2 mL/mmol). This was cooled to 0 °C before adding 2.0 M isopropylmagnesium chloride in THF (6.30 mL, 12.5 mmol, 2.40 eq). The white suspension was stirred for 1 h at 0 °C to generate the magnesium salt **5** to be used for the next reaction.

To vial #2 was added (*S*)-2-((*tert*-butoxycarbonyl)amino)-5-methoxy-5-oxopentanoic acid **6a** (1.4 g, 5.2 mmol, 1.0 eq) and THF (25 mL). The solution was cooled to 0 °C before addition of 1,1′-carbonyldiimidazole (0.85 g, 5.2 mmol, 1.0 eq). The reaction was stirred for 1 h at 0 °C. After 1 h vial #2 was cooled to −20 °C followed by dropwise addition of magnesium salt **5** from vial #1. The mixture was stirred for 45 min at −20 °C, and then allowed to warm to room temperature and stir for 3.5 h. The reaction contents were then poured onto 1.0 M HCl, extracted with EtOAc, washed in succession with saturated sodium bicarbonate and brine then dried over sodium sulfate. The organic layer was then filtered and concentrated under reduced pressure to provide crude 1-benzyl 7-methyl (4*S*)-4-((tert-butoxycarbonyl)amino)-2-fluoro-3-oxoheptanedioate **7a**. 

The residue was dissolved in toluene and Pd/C (0.10 g, 0.94 mmol, 0.18 eq) was added followed by bubbling with hydrogen gas through the solution for 10 min. The bubbling was stopped and the reaction was allowed to stir under hydrogen atmosphere overnight at room temperature. The mixture was then filtered, concentrated, and purified via flash chromatography (5–60% EtOAc in hexanes) to yield **8a** (0.85 g, 3.1 mmol, 59%) as a white solid. ^1^H NMR (500 MHz, Chloroform-d) δ 5.21 (s, 1H), 5.15–4.91 (m, 2H), 4.62 (s, 1H), 3.68 (s, 3H), 2.57–2.31 (m, 2H), 2.27–2.16 (m, 1H), 1.88 (dq, *J* = 14.8, 7.4 Hz, 1H), 1.44 (s, 9H). APCI-MS: *m*/*z* 278.0 [M + H]^+^. 

##### Methyl ((benzyloxy)carbonyl)-L-phenylalanyl-L-alaninate (**12g**)

To a solution of ((benzyloxy)carbonyl)-*L*-phenylalanine (0.72 g, 2.4 mmol, 1.0 eq) and *N*-methylmorpholine (0.97 g, 9.6 mmol, 4.0 eq) in anhydrous THF was added isobutyl chloroformate (0.49 g, 3.6 mmol, 1.5 eq) at 0 °C. The methyl *L*-alaninate hydrochloride (0.33 g, 2.4 mmol, 1.0 eq) was then added and the reaction was allowed to warm slowly to room temperature overnight. The next day the reaction mixture was diluted with EtOAc (25 mL) and washed in succession with 1.0 M HCl (50 mL), 5% sodium carbonate solution (50 mL), brine (50 mL), then dried over sodium sulfate before filtering and concentrating *in vacuo*. The residue was recrystallized in a DCM:Hexanes solution and filtered, yielding **12g** as a white crystalline solid (0.61 g, 1.6 mmol, 66%). ^1^H NMR (500 MHz, DMSO-*d*_6_) δ 8.49 (d, *J* = 7.1 Hz, 1H), 7.49 (d, *J* = 8.8 Hz, 1H), 7.37–7.02 (m, 10H), 4.88 (s, 2H), 4.39–4.10 (m, 2H), 3.58 (s, 3H), 2.96 (dd, *J* = 13.9, 3.7 Hz, 1H), 2.67 (dd, *J* = 13.7, 11.0 Hz, 1H), 1.28 (d, *J* = 7.3 Hz, 3H); APCI-MS: *m/z* 385.1 [M + H]^+^.

##### ((Benzyloxy)carbonyl)-L-phenylalanyl-L-alanine (**13g**)

The dipeptide methyl ester **12g** (0.61 g, 1.6 mmol, 1.0 eq) was dissolved in THF:H_2_O (4:1, 10 mL) followed by addition of LiOH (0.046 g, 1.9 mmol, 1.2 eq) at 0 °C. The mixture was stirred at 0 °C for 3 h before acidifying with 1.0 M HCl (2 mL) to < pH 3. The aqueous phase was extracted with EtOAc (3 × 5 mL) and the organic layers were combined and dried over sodium sulfate, filtered, and concentrated to yield **13g** as a white solid (0.52 g, 1.4 mmol, 87%) which was taken forward crude to the next step.

##### Cbz-L-Phe-L-Ala-L-Glu(OMe)-fluoromethylketone (**21**)

To vial #1 was added **8a** (0.050 g, 0.18 mmol, 1.0 equiv) and 4.0 M HCl in 1,4-dioxane (1.0 mL, 4.0 mmol, 22 eq). This was allowed to stir for 30 min at room temperature and monitored for completion by TLC, which indicated the formation of a very polar product. Upon completion, the solvent was removed in vacuo, and the oil was triturated with diethyl ether for 10 min and carefully decanted and dried in vacuo. The resulting Boc-removed Glu(OMe)-fluoromethylketone oil was diluted in THF and used directly in the next step.

To vial #2 was added **13g** (0.054 g, 0.14 mmol, 0.8 eq) in THF under argon atmosphere. To this solution was added isobutyl chloroformate (0.036 mL, 0.27 mmol, 1.5 eq) and *N*-methylmorpholine (0.079 mL, 0.72 mmol, 4.0 eq) at 0 °C under argon atmosphere. This was allowed to stir for 45 min at 0 °C after which the deprotected Glu(OMe)-fluoromethylketone in THF from vial #1 was added dropwise at 0 °C and allowed to stir for 15 min before warming to room temperature slowly overnight. The next day the reaction was diluted with EtOAc and washed in succession with 1 N HCl, 5% sodium bicarbonate, and brine. The organic layer was collected and dried over sodium sulfate, filtered, and concentrated in vacuo to provide the crude product. This residue was then recrystallized in DCM:Hexanes overnight, filtered, and dried in vacuo to yield **21** as a white solid (0.033 g, 0.063 mmol, 35%). ^1^H NMR (800 MHz, DMSO-*d*_6_) δ 8.33 (d, *J* = 7.4 Hz, 1H), 8.27 (d, *J* = 6.8 Hz, 1H), 7.51 (d, *J* = 8.5 Hz, 1H), 7.35–7.14 (m, 11H), 5.25–5.07 (m, 2H), 4.93 (s, 2H), 4.40–4.15 (m, 3H), 3.57 (s, 3H), 3.01 (dd, *J* = 14.0, 3.7 Hz, 1H), 2.78–2.64 (m, 1H), 2.40–2.26 (m, 2H), 2.12–1.98 (m, 1H), 1.86–1.63 (m, 1H), 1.26 (d, *J* = 7.1 Hz, 3H). ^13^C NMR (200 MHz, DMSO-*d*_6_) δ 203.7 (d, *J* = 14 Hz), 173.3, 173.1, 172.0, 156.3, 138.5, 137.4, 129.6, 128.7, 128.4, 128.1, 127.8, 126.6, 84.3 (d, *J* = 178 Hz), 65.6, 56.3, 54.7, 51.8, 48.7, 37.7, 29.7, 24.6, 18.0. ESI-MS: *m/z* 530.0 [M + H]^+^; HPLC *t*_R_ = 11.974 min (Column B); HPLC Purity: 98.2%.

### 3.2. Biological Evaluation

#### 3.2.1. Fluorescence-based Ub-Rho Deubiquitinase Activity Assay

The assay was performed in black 384-well plates (Catalog #12566624, Fisher, Waltham, MA, USA). DUB stock solutions were diluted in reaction buffer (50 mm Tris pH 7.6, 0.5 mm EDTA, 5 mm DTT, 0.1 % (*w*/*v*) BSA) to a concentration of 2.5 nM for UCHL1 or 0.025 nM for UCHL3. Stock solutions of Ub-Rhodamine 110 (U-555, Boston Biochem, Cambridge, MA, USA); 125 nM for UCHL1 assay, and 250 nM for UCHL3 assay) were prepared in the same buffer. A 10 mM stock solution was made for each inhibitor in DMSO, then a dilution of 600 µM in reaction buffer was made followed eight by 1:1 serial dilutions. To each well was added 20 µL of DUB stock solution and 10 µL of inhibitor solutions for nine final inhibitor concentrations of ranging from 0.78 µM–200 µM along with a DMSO only control well. These were allowed to incubate, while sealed, for the 3 h at room temperature. After incubation 20 µL of each Ub-Rho stock solution was added to the respective wells for each DUB to yield final concentration of 50 nM for UCHL1 or 100 nM for UCHL3, respectively. Plates were read immediately and fluorescence of cleaved Rhodamine 110 fluorophore was monitored at λ_ex_ = 485 nm, λ_em_ = 535 nm continuously for 20 min on a Synergy Neo2 instrument (BioTek, Winooski, VT, USA) The raw data was loaded into GraphPad Prism 8 (GraphPad Software, San Diego, CA, USA; www.graphpad.com, accessed on 12 January 2021) and the slope of the linear portion of the fluorescence vs. time curves was calculation for each inhibitor concentration and % activity of the enzyme was determined compared to DMSO-treated controls. The % activity was plotted as a function of inhibitor concentration and the data was fitted with non-linear regression analysis to calculate the IC_50_ values. Errors are reported as the 95% confidence interval calculated from experiments collected in technical triplicate. 

#### 3.2.2. Time-Dependent Inhibitor Analysis

This assay was performed the same as described above with the exception that instead of 3-h pre-incubation followed by Ub-Rho addition the Ub-Rho substrate was added at 20-min increments following addition of inhibitor to wells beginning at 0 min and ending at 120 min. Plates were read continuously for 20 min following addition of Ub-Rho. The % activity of each inhibitor concentration was the plotted vs. time of pre-incubation and the slope of this plot is the pseudo first-order rate constant (*k*_obs_) for inhibition at each concentration. The *k*_obs_ was plotted as a function of inhibitor concentration and then fit to the equation Y = *k*_inact_X/(*K*_I_ + X) to calculate *k*_inact_, *K*_I_ and *k*_inact_/*K*_I_ as described by Resnick et al. [64].

#### 3.2.3. Ub Binding Affinity Analysis Using Biolayer Interferometry

This assay was carried out according to our previously published protocol using an Octet RED384 biolayer interferometer (ForteBio, Fremont, CA, USA) [69]. A solution containing 5 µM His-UCHL1 was incubated with 2 mM VAEFMK **1** or DMSO overnight in reaction buffer (50 mM Tris pH 7.6, 0.5 mM EDTA, 5 mM DTT) at room temperature before buffer exchanging into water using Zeba spin desalting columns (Thermo Scientific, catalog no. 89882). The concentration of His-UCHL1 was determined by A_280_ on a NanoDrop system (Thermo Scientific), after which His-UCHL1 was diluted into BLI buffer [1× PBS containing 0.05% (*v*/*v*) Tween 20 and 0.1% (*w*/*v*) bovine serum albumin (BSA)]. The concentration of Ub was determined by the BCA assay and diluted to top concentrations into BLI buffer, and 1:1 serial dilutions were completed. The top concentration of Ub was 2 μM. 40 μL of each solution was added to a 384-well tilted-bottom plate (part no. 185080, Molecular Devices, San Jose, CA, USA). The Ni-NTA biosensor was dipped first into BLI buffer (initial baseline, 60 s), then into the His-UCH protein wells (loading step, 300 s), then into BLI buffer alone (baseline step, 60 s), then into the lowest concentration of UbV (association step, 120 s), and then into buffer alone (dissociation step, 100 s). A reference sensor loaded with protein was dipped into wells containing only buffer to adjust for protein-buffer signals. The association-dissociation was repeated with increasing concentrations of UbV. All measurements were taken at 30 °C. 

Octet RED384 Data Analysis Software (ForteBio, version 9.0.0.15) was used to collect and analyze the raw data for the association and dissociation curves. After subtraction of a reference sensor (loaded sensors dipped into wells containing only buffer), averages of the association responses (in nanometer response signal from 110 to 115 s) were calculated and plotted as a function of UbV concentration in Prism 8. These data were fit to a nonlinear regression one-site specific binding model to determine a K_d_.

#### 3.2.4. Protein Expression and Purification

UCHL1, UCHL1^C90A^, and UCHL3 (Expressed in pET15b by GenScript) for biochemical assays were grown in LB growth medium at 37 °C to an optical density of 0.6–0.8. After 0.1 mm IPTG induction at 17 °C for 18 h the bacteria were lysed and pelleted at 15,000g, and clarified lysate was purified on HisPur Ni-NTA resin (Thermo Scientific) according to manufacturer’s instructions.

#### 3.2.5. Copper-Catalyzed Click Reaction and In-Gel Fluorescence for Wild-Type and C90A UCHL1

Solutions containing 1 mg/mL recombinant UCHL1^WT^ or UCHL1^C90A^ (from pET15b) (49 µL) was incubated with 10 µM inhibitor for 3 h at room temperature in PBS starting block buffer (Thermo Scientific # 37538). After 3 h, 6 µL of a freshly prepared cocktail was added to each sample, consisting of: 3 µL 1.7 mM THPTA in 1:4 DMSO/tBuOH (100 µM final concentration), 1 µL 50 mM CuSO_4_ (1 mM final concentration), 1 µL TCEP HCl (25 µM final concentration), and 1 uL Cy5-Azide (#AZ118, Click Chemistry Tools, Scottsdale, AZ, USA; 25 µM final concentration). The samples were allowed to react for 2 h at room temperature protected from light before precipitating the sample by adding 600 µL MeOH, 150 µL chloroform, and 400 µL water, vortexing between each addition. The samples were pelleted by centrifuging at 170,000× *g* for 5 min. The upper aqueous layer was removed without disturbing the precipitate, and 450 µL MeOH was added again, followed by centrifuging at 17,000× *g* for 5 min and decanting the solvent (2X). The samples were allowed to air dry for 30 min before resuspending in 1× Laemmli buffer and boiling at 90 °C for 5 min. The samples were then analyzed by SDS-PAGE and imaged on an Odyssey system (LI-COR, Lincoln, NE, USA) and subsequently Coomassie stained.

#### 3.2.6. Mass Spectrometry Analysis of 34 Adducts on UCHL1

Solutions containing 10 µM recombinant UCHL1^WT^ or UCHL1^C90A^ (from pET15b) (49 µL) was incubated with either 10 µM or 20 µM inhibitor for 3 h at room temperature in PBS starting block buffer (Thermo Scientific # 37538). To quench reaction, chilled acetone was added to the sample to achieve an overall 80% acetone solution. After overnight storage at −20 °C, suspension was centrifuged at 14,000× *g* at 4 °C for 15 min. Supernatant was discarded, and pellet was dried by vacuum centrifugation for 1 h. Pellet was reconstituted in 50/50 water/acetonitrile with 0.1% formic acid. 25 pmol per sample was analyzed by LC/MS (Agilent 1260 Infinity II with a ZORBAX Rapid Resolution High Definition 300Å Stable Bond C3, 2.1 × 100 mm, 1.8 µm column) attached to an Agilent 6129 quadrupole mass spectrometer in positive ion mode.

The column was held at 45 °C. Mobile solution A was 0.1% formic acid in water, and mobile phase B was 0.1% formic acid in acetonitrile. The gradient used was hold at 5% B for 5 min, increase linearly to 100% B for 10, and then hold at 95% B for 5 min. The mass data were collected at a range of 500–1000 *m*/*z*.

Raw data were processed using MestReNova. For all samples, the deconvoluted mass was calculated with a charged state range from 27 to 36, *m*/*z* range of 670–900 Da, and deconvoluted mass range from 25,000–26,000 Da. Representative data had an abundance threshold of 10–20% for charged state deconvolution calculation. For C90A mutant, this abundance threshold was lowered to 5% to detect the presence of any reacted protein. **34** adduct is expected to be +486.00 Da after displacement of fluorine.

#### 3.2.7. KMS Cell Proliferation Assay

The proliferation of KMS11 and KMS12 cells was monitored using an IncuCyte devise (Sartorius, Goettingen, Germany; www.essenbioscience.com, accessed on 31 August 2020). Cells (3 × 10^4^ per well; 200 µL) were seeded in triplicate in 96 well plates the night prior to the addition of compounds. The IncuCyte was set to collect images from five fields per well at 4-h intervals by using the nuclear dye Nuclight Red (Sartorius). At each timepoint, the number of cells per well was deter- mined by taking the mean of the five locations in each well. The relative proliferation at each timepoint was normalized, with 100% set as the number of cells observed for incubated with DMSO (0.1%) controls at 72 h.

#### 3.2.8. KMS Cell Activity-Based Probe Target Engagement Assay

Twelve hours prior to the addition of compounds to the cells, 1.2 million KMS11 or KMS12 cells mL^−1^ per well were seeded in a 12 well plate. Cells were incubated with inhibitor for 4 h before washing the cells three time with PBS to remove excess compound. Cells were harvested, pelleted, and lysed in 60 µL of lysis buffer (0.5%NP-40 in 50 mM Tris pH 7.4). HA-Ub-VME was then added to a final concentration of 1 µM and incubated for 1 h at room temperature. An equal volume of 2X Laemmli sample buffer was added, and the samples were heated to 95 °C before separating by gel electrophoresis and analyzed by immunoblot.

#### 3.2.9. SW1271 Cell Viability Assay

Luciferase-constructed cancer cells were seeded in 96-well plates at 10,000 cells/well with the compounds at the indicated concentrations. DMSO was used as vehicle control. Luminescence readings were acquired after adding 1% D-luciferin (GoldBio, St. Louis, MO, USA) after 24 h. Cell viability was determined by the ratio of luminescence reading compared to the one at 0 h, and normalized to vehicle control. 

#### 3.2.10. SW1271 Cell Activity-Based Probe Target Engagement Assay

Cells were treated with 100 µM inhibitor or DMSO for 4 h before being washed, scraped, and collected. Cell pellets were thawed on ice before resuspending in 200 µL lysis buffer (50 mM Tris pH 7.6, 150 mM NaCl, 5 mM MgCl_2_, 0.5 mM EDTA, 5 mM DTT, 2 mM ATP, 0.5% NP-40, 10% *v*/*v* glycerol) and incubating on ice for 30 min with vigorous vortexing every 10 min. Cell lysate was clarified by centrifugation (17,000× *g* for 10 min) and the supernatant was collected, and the concentration was determined by Bradford (performed according to manufacturer’s protocol). Cells were normalized to 2 mg mL^−1^. To each reaction mixture was added 36 µL of lysate and 2 µL of HA-Ub-VME or lysis buffer. This was incubated for 30 min at room temperature before quenching with 15 µL of 4X Laemmli buffer and heating to 95 °C for 5 min. Samples were subjected to SDS-PAGE and immunoblot.

#### 3.2.11. Copper-Catalyzed Click Reaction and In-Gel Fluorescence of Treated SW1271 Cells

Cells were treated with 100 µM inhibitor or DMSO for 4 h before being washed, scraped, and lysed in 200 µL of lysis buffer (1× PBS, 1% *v*/*v* Triton X-100, 0.1% *w*/*v* SDS, 1X HALT) for 30 min on ice with vigorous vortexing every 10 min. Samples were then pelleted at 17,000× *g* for 10 min before collecting the supernatant and measuring concentration using BCA. Samples were normalized to 2 mg/mL and stored at −80 °C overnight. The following day, samples were thawed. While thawing, a click cocktail was prepared (for each sample, the cocktail consists of: 3 µL of 1.7 mM THPTA in 1:4 tBuOH/DMSO, 1 µL of 50 mM CuSO4 in water, 1 µL of 1.25 mM Cy5-N3 in DMSO, and 1 µL of 50 mM TCEP in water freshly prepared). 50 µL of each sample was added to a new tube, and 6 µL of click cocktail was also added to these tubes. The samples were allowed to react for 2 h at room temperature protected from light before precipitating the sample by adding 600 µL MeOH, 150 µL Chloroform, and 400 µL water, vortexing between each addition. The samples were pelleted by centrifuging at 17,000× *g* for 5 min. The upper aqueous layer was removed without disturbing the precipitate, and 450 µL MeOH was added again, followed by centrifuging at 17,000× *g* for 5 min (2X) and decanting the solvent. The samples were allowed to air dry for 30 min before resuspending in 4X Laemmli buffer and boiling at 90 °C for 5 min. The samples were then analyzed by SDS-PAGE and imaged on a Licor Odyssey and subsequently Coomassie stained.

#### 3.2.12. shRNA UCHL1 Knockdown in SW1271 Cells

pLKO.1 TRC vectors with shRNAs targeting human UCHL1 were packaged into lentiviral particles and used for stable transduction of the SW1271 cells via puromycin selection. The shRNA sequences are listed in Supplementary Table S3. The pLKO.1 vector with non-targeting, scrambled shRNA was used as a control. Depletion of UCHL1 was determined via immunoblotting.

#### 3.2.13. Scratch Wound Healing Cell Migration Assay

Wild-type or shRNA treated UCHL1 knockdown SW1271 cells were seeded in 12-well plates at 1 million cells/well. The cells were cultured overnight to grow as a confluent single layer. The cross-style wounds were created using pipet tips to scratch on the single layer of cells. The compounds were prepared in full growth media at the desired concentrations and added after the wounds were created. DMSO was used as the vehicle control. Photos of the wounds were captured immediately after adding treatments at 0 h. Photos of the wounds were captured at 0 and 24 h. The areas of cross-style wounds were quantified using Image J 1.52a software. The migration rate (%) was defined as the percentage of area at each timepoint compared to the one at 0 h.

## 4. Conclusions

In summary, we have presented an SAR study and biological characterization of an irreversible covalent inhibitor of UCHL1. Starting with the parent compound VAEFMK **1** it was determined that bulky, lipophilic moieties are preferred in the valine position, while alteration of the alanine position was not tolerated. Additionally, it was determined that unnatural *D*-amino acids abrogate activity. The SAR at these two positions could be rationalized by observations of the VAEFMK-bound UCHL1 crystal structure. Installing an alkyne handle to the benzyloxy capping group of the peptide improved activity when compared to three nearest neighbor analogs suggesting future SAR work on the benzyloxy may improve potency. To round out SAR it was determined that modification of the fluoromethylketone electrophilic site to a more reactive chloromethylketone surprisingly lost activity versus UCHL1 in vitro. Furthermore, when the chloromethylketone derivative was tested in cellular assays it was toxic and shown to non-specifically label numerous proteins. Conversely, the fluoromethylketones were selective for only UCHL1 over the closely related UCHL3. Ultimately, the scaffold was improved from a UCHL1 IC_50_ value of 24 µM for VAEFMK **1** to 7.7 µM for **34** with no activity against the closest related UCHL3 and only slight inhibition of UCHL5 at 250 µM. Finally, the probes were shown to engage UCHL1 selectively in two different cancer relevant cells lines. Molecule **1** displayed anti-proliferative properties versus KMS11 myeloma cells dependent on UCHL1 while the compound displayed no effect on KMS12 cells that do not express UCHL1. Analog **34** displayed anti-migratory properties in SW1271 small cell lung cancer cell lines consistent with observed behavior from genetic depletion of UCHL1. While the probe displays modest potency toward UCHL1 it is quite selective in cells and allowed for probing UCHL1 without reduction in cell viability as opposed to the shRNA knockdowns, which displayed some reduced viability. A potential way to improve the cellular activity of the scaffold may be to alter the Glu(OMe) moiety. This ester is likely cleaved by cellular esterases leaving the carboxylate side chain, which was shown to be inactive *in vitro*. The cellular inhibition is likely a competition between inhibitor engagement with UCHL1 and the ester being cleaved. Further optimization of this residue is ongoing. Alternative UCHL1 inhibitors from the cyanopyrrolidine scaffold, although selective *in vitro*, still exhibit off-target toxicity in the micromolar range while **34** does not exhibit any cellular toxicity up to 100 µM. Analog **34** is yet another option to selectively probe UCHL1 and offers additional capabilities in that an alkyne is already installed to be leveraged for various chemical biology applications.

## Figures and Tables

**Table 1:**
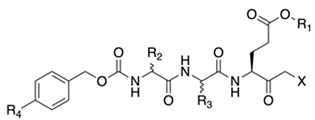
UCHL1 inhibition data for VAEFMK analogs.

Cpd	X	R_1_	^a^ R_2_	^a^ R_3_	R_4_	IC_50_ (µM) ^b^
**1**	F	Me	Val	Ala	H	24
**14**	Cl	Me	Val	Ala	H	>200
**15**	F	Me	Val	Gly	H	95
**16**	F	H	Val	Gly	H	>200
**17**	F	Me	*D*-Val	Ala	H	>200
**18**	F	Me	Gly	Ala	H	>200
**19**	F	Me	Ala	Ala	H	76
**20**	F	Me	Leu	Ala	H	23
**21**	F	Me	Phe	Ala	H	13
**22**	F	Me	Ser	Ala	H	185
**23**	F	Me	Thr	Ala	H	100
**24**	F	Me	Asn	Ala	H	119
**25**	F	Me	Asp	Ala	H	>200
**26**	F	Me	Val	*D*-Ala	H	>200
**27**	F	Me	Val	Leu	H	>200
**28**	F	Me	Val	Phe	H	>200
**29**	F	Me	Val	Ser	H	100
**30**	F	Me	Val	Thr	H	>200
**31**	F	Me	Val	Asn	H	>200
**32**	F	Me	Val	Asp	H	>200
**33**	F	Me	Val	Glu	H	>200
**34**	F	Me	Val	Ala	-C≡CH	7.7
**35**	F	Me	Val	Gly	-C≡CH	62
**36**	F	Me	Phe	Ala	-C≡CH	11
**37**	Cl	Me	Val	Ala	-C≡CH	>200

^a^ Amino acids are *L* unless otherwise specified as *D*. ^b^ Experiments were performed in technical triplicate and averages are reported. IC_50_ values are from 3 h of preincubation with UCHL1. Errors provided as 95% confidence intervals reported in supporting material Table S1.

**Table 2:** Observed deconvoluted masses for recombinant WT- and C90A-UCHL1 treated with **34**.

Protein and Treatment	Calculated Deconvoluted Masses Observed (Da) ^a^	Adduct(s) Observed (Da)
UCHL1	25,235.782	none
WT-UCHL1 + **34** (1:1)	25,235.782 and 25,721.782	+486.00
WT-UCHL1 + **34** (1:2)	25,235.782 and 25,721.782	+486.00
UCHL1 C90A	25,205.782	none
UCHL1 C90A + **34** (1:1)	25,205.782	none
UCHL1 C90A + **34** (1:2)	25,205.782	none

^a^ Average of duplicate samples.

**Table 3:** Kinetics of covalent inhibition of UCHL1.

Cpd	*k*_inact_ (s^−1^)	*K*_I_ (µM)	*k*_inact_/*K*_I_ (M^−1^s^−1^)	R^2^
**1**	0.0173	60.04	288.1	0.9623
**34**	0.0136	16.79	810.0	0.9779
**36**	0.0157	75.32	208.4	0.9478

## Data Availability

Data is contained within the article or Supplementary Material.

## Supplementary Materials

The following are available online, Scheme S1: Synthesis of p-ethynyl-benzyloxy-protected dipeptides, Table S1: UCHL1 inhibition provided as 95% confidence interval, Figure S1: Dose-response curves for analogs **1** (A) and **34** (B) against UCHL3 Figure S2: Proposed cyclization of glutamate-fluoromethylketone, Figure S3: Dark-exposure HA-immunoblot data for KMS11, Table S2: Cell lines and culture conditions, Table S3: Detailed information for shRNA vectors. Synthetic procedures and characterization of molecules also provided.

## Sample Availability

Samples of the compounds are available from the corresponding author upon request.
