# Supplementary material for: Optimization and Anti-Cancer Properties of Fluoromethylketones as Covalent Inhibitors for Ubiquitin C-Terminal Hydrolase L1
Source: Molecules. 2021 Feb 25;26(5):1227. doi: 10.3390/molecules26051227 (PMC7956625; doi:10.3390/molecules26051227)
Supplement: Supplementary file 1 [file molecules-26-01227-s001.pdf]

Supplementary Material

Optimization and Anti-Cancer Properties of Fluoromethylketones as Covalent Inhibitors for Ubiquitin C-Terminal Hydrolase L1

Aaron D. Krabill[1], Hao Chen[1], Sajjad Hussain[2,3], Chad S. Hewitt[1], Ryan D. Imhoff[1], Chittaranjan Das[4,5], Paul J. Galardy[2], Michael K. Wendt[1,5,6] and Daniel P. Flaherty [1,5,6,*]

[1] Department of Medicinal Chemistry and Molecular Pharmacology, College of Pharmacy, 575 Stadium Mall Dr. West Lafayette, IN 47907; akrabil@purdue.edu (A.D.K.), chen2334@purdue.edu (H.C.), hewitt8@purdue.edu (C.S.H.), rimhoff@purdue.edu (R.D.I.), mwendt@purdue.edu (M.K.W.), dflaher@purdue.edu (D.P.F.)

[2] Division of Pediatric Hematology-Oncology, Mayo Clinic, 200 First St. Guggenheim 15, Rochester, MN 55905; hussain.sajjad@mayo.edu (S.H.), galardy.paul@mayo.edu (P.J.G.)

[3] Department of Pediatric and Adolescent Medicine, Mayo Clinic, 200 First St. Guggenheim 15, Rochester, MN 55905.

[4] Department of Chemistry, College of Science, 560 Oval Dr. West Lafayette, IN 47907; cdas@purdue.edu (C.D.)

[5] Purdue Center for Cancer Research, Hanson Life Sciences Research Building, 201 University St. West Lafayette, IN, 47907

[6] Purdue Institute for Drug Discovery, 720 Clinic Ln. West Lafayette, IN 47907

* Correspondence: dflaher@purdue.edu (D.P.F.)

Table of Contents:

Scheme S1. Synthesis of p-ethynyl-benzyloxy-protected dipeptides……………………………………pg 2.

Table S1. UCHL1 inhibition provided as 95% confidence interval………………………………………pg 2.

Figure S1. Dose-response curves for analogs **1** (A) and **34** (B) against UCHL3……………………………pg 3.

Figure S2. Proposed cyclization of glutamate-fluoromethylketone………………………………………pg 3.

Figure S3. Dark-exposure HA-immunoblot data for KMS11………………………………………………..pg 4.

Table S2. Cell lines and culture conditions…………………………………………………………………pg 4.

Table S3. Detailed information for shRNA vectors…………………………………………………………..pg 4.

Synthetic Procedures and Characterization of Molecules………………………………………………pg 5 – 16.

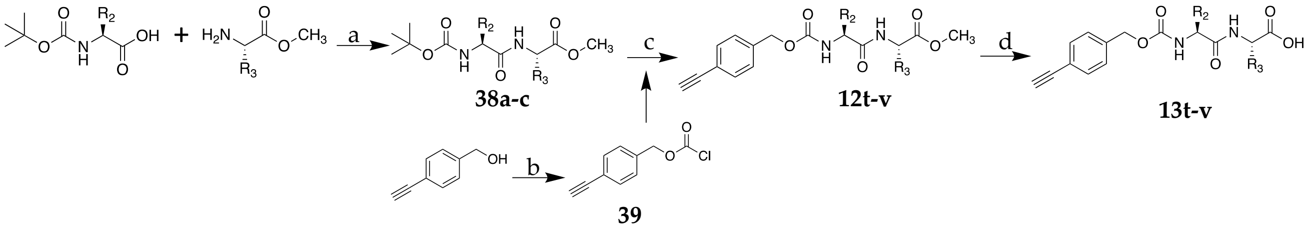

Scheme S1. Synthesis of p-ethynyl-benzyloxy-protected dipeptides. Reagents and conditions: a) Boc-carboxylic acid (1.0 eq), methyl ester amino acid (1.0 eq), *N*-methylmorpholine (4.0 eq), isobutyl chloroformate (1.5 eq), THF, 0 °C – RT, overnight; b) 4-ethynylbenzyl alcohol (1.0 eq), triphosgene (1.2. eq), triethylamine (2.0 eq). c) i. **38 a – c** (1.0 eq), 4.0 M HCl in 1,4-dioxane (11 eq), 30 minutes, RT; ii. **39** (1.01 eq), triethylamine (2.4 eq), THF, 0 °C – RT, overnight; d) LiOH (1.2 eq), 4:1 THF:H_2O, 0 °C, 3 hours.

Table S1. UCHL1 inhibition data for VAEFMK analogs

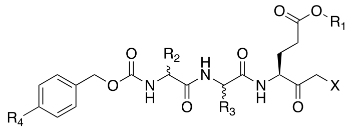

Cpd	X	R_1	[a]R_2	[a]R_3	R_4	IC_{50} (μM)[b]
**1**	F	Me	Val	Ala	H	18.4 to 31.8
**14**	Cl	Me	Val	Ala	H	n.d.
**15**	F	Me	Val	Gly	H	73.3 to 125.0
**16**	F	H	Val	Gly	H	n.d.
**17**	F	Me	*D*-Val	Ala	H	n.d.
**18**	F	Me	Gly	Ala	H	n.d.
**19**	F	Me	Ala	Ala	H	53.6 to 83.8
**20**	F	Me	Leu	Ala	H	16.6 to 32.0
**21**	F	Me	Phe	Ala	H	10.0 to 17.9
**22**	F	Me	Ser	Ala	H	160 to 220
**23**	F	Me	Thr	Ala	H	80.3 to 125
**24**	F	Me	Asn	Ala	H	86.5 to 168
**25**	F	Me	Asp	Ala	H	n.d.
**26**	F	Me	Val	*D*-Ala	H	n.d.
**27**	F	Me	Val	Leu	H	n.d.
**28**	F	Me	Val	Phe	H	n.d.
**29**	F	Me	Val	Ser	H	76.9 to 131.5
**30**	F	Me	Val	Thr	H	n.d.
**31**	F	Me	Val	Asn	H	n.d.
**32**	F	Me	Val	Asp	H	n.d.
**33**	F	Me	Val	Glu	H	n.d.
**34**	F	Me	Val	Ala	-C≡CH	6.1 to 9.7
**35**	F	Me	Val	Gly	-C≡CH	48.5 to 79.6
**36**	F	Me	Phe	Ala	-C≡CH	8.93 to 13.8
**37**	Cl	Me	Val	Ala	-C≡CH	n.d.

[a]Amino acids are *L* unless otherwise specified at *D*. [b]Experiments were performed in technical triplicate and averages are reported. IC_{50} values are from 3 hours of preincubation with UCHL1. Values provided as 95% confidence intervals

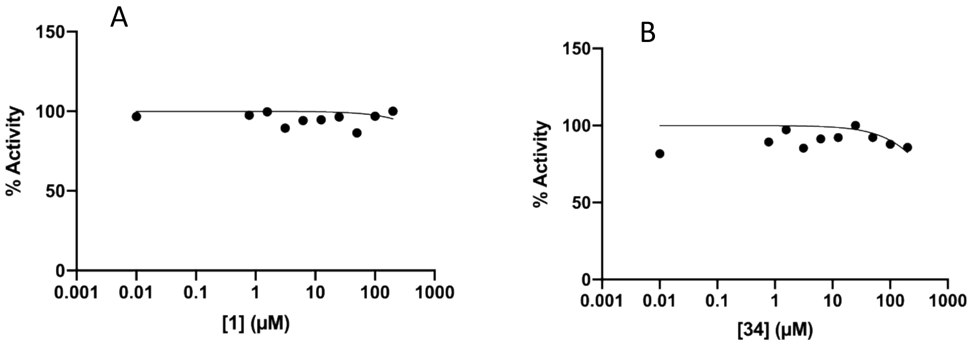

Figure S1. Dose-response curves for analogs **1** (A) and **34** (B)against UCHL3.

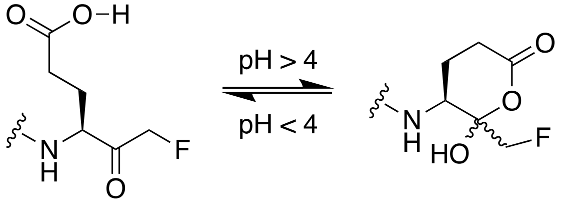

Figure S2. Proposed lactone cyclization for analog **16** based on previously reported cyclic lactone formation for aspartate-fluoromethylketone molecules.

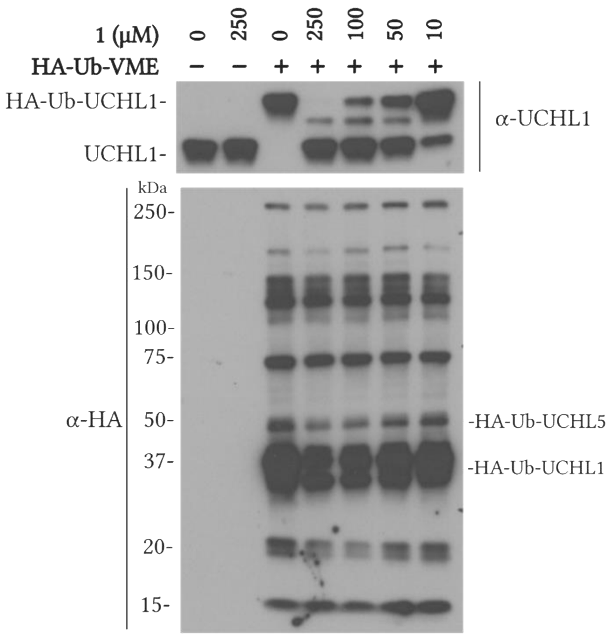

Figure S3. Dark-exposure HA-immunoblot data for KMS11 cells treated with **1** (0 – 250 μM) then reacted with HA-Ub-VME. Blotting for UCHL1 (top panel) indicates the molecular weight shift of the HA-Ub-UCHL1 complex in the control lane and the dose-dependent decrease of this complex formation in KMS11 cells. Blotting for HA (bottom panel, dark exposure) confirms only dose-dependent decrease of HA-Ub-UCHL1 complex formation and no dose-dependent decrease of any other DUB.

Table S2. Cell Lines and Culture conditions

Name of cell line	Culture condition
SW1271 (ATCC® CRL-2177™)	DMEM with 10% Fetal Bovine Serum (FBS)
KMS11 (JCRB1179)	DMEM with 10% Fetal Bovine Serum (FBS)
KMS12-BM (JCRB0429)	DMEM with 10% Fetal Bovine Serum (FBS)

Table S3. Detailed information for shRNA vectors

Simplified ID	Source Clone ID	Target	Vector	Mature Antisense
079	TRCN0000011079	UCHL1 ORF	pLKO.1	AAAGAAACTGTTTCAGAACTG
274	TRCN0000007274	UCHL1 ORF	pLKO.1	ATTCACCTTGTCATCTACCCG

Synthetic Procedures and Characterization of Molecules

Tert-butyl (S)-4-((tert-butoxycarbonyl)amino)-6-fluoro-5-oxohexanoate (**8b**)

Synthesized according to same procedure for **8a** using **4** (1.3 g, 6.2 mmol, 1.3 eq), 2.0 M *iso*-propylmagnesium chloride in THF (6.2 mL, 12 mmol, 2.5 eq), (*S*)-5-(*tert*-butoxy)-2-((*tert*-butoxycarbonyl)amino)-5-oxopentanoic acid (1.5 g, 4.9 mmol,1.0 eq), and 1,1'-carbonyldiimidazole (0.84 g, 5.2 mmol, 1.0 eq), Pd/C (0.052 g, 0.49 mmol, 0.1 eq), H_2 gas to yield **8b** (1.1 g, 4.0 mmol, 82 %). ^{1}H NMR (500 MHz, Chloroform-d) δ 5.23 (d, J = 8.0 Hz, 1H), 5.16 – 4.90 (m, 2H), 4.66 – 4.49 (m, 1H), 2.41 – 2.27 (m, 2H), 2.15 (dq, J = 12.2, 6.7 Hz, 1H), 1.86 (dt, J = 14.6, 7.4 Hz, 1H), 1.53 – 1.35 (m, 18H). APCI-MS: *m/z* 320.2 $[M+H]^+$.

Methyl (S)-4-((tert-butoxycarbonyl)amino)-6-chloro-5-oxohexanoate (**11**).

Using an Aldrich® diazomethane-generator with System 45™ compatible connection, to the outer tube of was added **6a** (280 mg, 1.1 mmol, 1.0 eq) in THF. To this stirring solution at 0 °C was added N-methylmorpholine (150 µL, 1.4 mmol, 1.3 eq) and isobutyl chloroformate (160 µL, 1.3 mmol, 1.2 eq). This was allowed to react for 45 minutes. To the inner tube was added N,4-dimethyl-N-nitrosobenzenesulfonamide (500 mg, 2.3 mmol, 2.2 eq), carbitol (1.0 mL), and ether (1.0 mL). The system was closed, and 1.5 mL of 37% w/v KOH was added to initiate the reaction. After 2 hours, the reaction in the outer tube reaction had turned yellow and was transferred to a new vial where 4.0 M HCl in 1,4-dioxane was added dropwise until the yellow color disappeared. This was allowed to stir at 0 °C for 30 minutes before diluting with EtOAc and washing with sodium bicarbonate solution, water, and brine, then drying over sodium sulfate and concentrating. The resulting oil was purified by flash chromatography (10 → 75% EtOAc in Hexanes) yielding **11** (0.20 g, 62 %). ^{1}H NMR (500 MHz, Chloroform-d) δ 5.21 (d, J = 8.1 Hz, 1H), 4.62 – 4.48 (m, 1H), 4.32 (s, 2H), 3.69 (s, 3H), 2.53 – 2.36 (m, 2H), 2.31 – 2.15 (m, 1H), 2.00 – 1.83 (m, 1H), 1.64 – 1.39 (m, 9H). APCI-MS: *m/z* 294.0 $[M+H]^+$.

Methyl ((tert-butoxyycarbonyl)-L-valyl-L-alaninate (**38a**)

Prepared according to the same procedure as **12g** using (tert-butoxycarbonyl)-*L*-valine (1.0 g, 4.6 mmol, 1 eq), methyl *L*-alaninate HCl (0.64 g, 4.6 mmol, 1 eq), isobutyl chloroformate (0.63 g, 4.6 mmol, 1.0 eq) and triethylamine (0.93 g, 9.2 mmol, 2 eq) to produce **38a** (0.49 g, 1.6 mmol, 35%). ^{1}H NMR (500 MHz, Chloroform-*d*) δ 6.36 (s, 1H), 5.04 (s, 1H), 4.68 – 4.47 (m, 1H), 3.98 – 3.84 (m, 1H), 3.75 (s, 3H), 2.27 – 2.01 (m, 1H), 1.50 – 1.31 (m, 12H), 0.95 (dd, *J* = 25.0, 6.8 Hz, 6H); APCI-MS: *m/z* 303.2 $[M+H]^+$.

Methyl ((tert-butoxycarbonyl)-L-valyl-glycinate (**38b**)

Prepared according to the same procedure as **12g** using (tert-butoxycarbonyl)-*L*-valine (0.39 g, 1.8 mmol, 1 eq), methyl glycinate HCl (0.22 g, 1.8 mmol, 1 eq), isobutyl chloroformate (0.37 g, 2.7 mmol, 1.5 eq) and triethylamine (0.36 g, 3.6 mmol, 2 eq) to produce **38b** (0.30 g, 1.6 mmol, 90%). ^{1}H NMR (500 MHz, Chloroform-*d*) δ 6.46 (s, 1H), 5.02 (d, *J* = 8.5 Hz, 1H), 4.14 – 3.89 (m, 3H), 3.76 (s, 3H), 2.29 – 2.07 (m, 1H), 1.45 (s, 9H), 0.95 (dd, *J* = 26.7, 6.8 Hz, 6H); APCI-MS: *m/z* 289.2 $[M+H]^+$.

Methyl ((tert-butoxycarbonyl)-L-phenylalaninyl- L-alaninate (**38c**)

Prepared according to the same procedure as **12g** using (tert-butoxycarbonyl)-*L*-phenylalanine (1.0 g, 3.8 mmol, 1 eq), methyl alaninate HCl (0.53 g, 3.8 mmol, 1 eq), isobutyl chloroformate (0.77 g, 5.7 mmol, 1.5 eq) and N-methylmorpholine (1.5 g, 15 mmol, 4 eq) to produce **38c** (0.89 g, 2.5 mmol, 67%). ^{1}H NMR (500 MHz, DMSO-d_6) δ 8.36 (d, *J* = 7.1 Hz, 1H), 7.29 – 7.11 (m, 5H), 6.87 (d, *J* = 8.8 Hz, 1H), 4.26 (p, *J* = 7.2 Hz, 1H), 4.19 – 4.09 (m, 1H), 3.58 (s, 3H), 2.93 (dd, *J* = 13.9, 3.9 Hz, 1H), 2.66 (dd, *J* = 13.9, 10.7 Hz, 1H), 1.30 – 1.20 (m, 11H); APCI-MS: *m/z* 351.3 [M+H]$^+$.

p-ethynylbenzyl carbonochloridate (**39**)

Triphosgene (1.4 g, 4.55 mmol, 1.2 eq) was dissolved in DCM. A mixture of (4-ethynylphenyl)methanol (0.5 g, 3.8 mmol, 1.0 eq) and triethylamine (1.05 mL, 7.6 mmol, 2.0 eq) in DCM was added dropwise to the stirring solution of triphosgene at 0 °C. After 30 minutes stirring at 0 °C the reaction mixture was evaporated to dryness to yield crude **39** (0.64 g, 3.3 mmol, 86%). ^{1}H NMR (500 MHz, Chloroform-*d*) δ 7.49 (d, *J* = 8.3 Hz, 2H), 7.35 (d, *J* = 8.2 Hz, 2H), 4.57 (s, 2H), 3.10 (s, 1H).

Synthesis of Cbz-protected dipeptide methyl esters **12.**

Methyl ((benzyloxy)carbonyl)-D-valyl-L-alaninate (**12c**)

Prepared according to the same procedure as **12g** using benzyloxycarbonyl-*D*-valine (0.60 g, 2.4 mmol, 1 eq), methyl *L*-alaninate HCl (0.33 g, 2.4 mmol, 1 eq), isobutyl chloroformate (0.49 g, 3.6 mmol, 1.5 eq) and N-methylmorpholine (0.97 g, 9.6 mmol, 4 eq) to produce **12c** (0.81 g, 2.0 mmol, 84%). ^{1}H NMR (500 MHz, DMSO-d_6) δ 8.31 (d, *J* = 7.1 Hz, 1H), 7.43 – 7.14 (m, 6H), 5.00 (d, *J* = 1.4 Hz, 2H), 4.23 (p, *J* = 7.2 Hz, 1H), 3.86 (dd, *J* = 9.3, 6.9 Hz, 1H), 3.58 (s, 3H), 1.99 – 1.76 (m, 1H), 1.22 (d, *J* = 7.3 Hz, 3H), 0.80 (t, *J* = 7.0 Hz, 6H); APCI-MS: *m/z* 337.2 [M+H]$^+$.

Methyl ((benzyloxy)carbonyl)-glycyl-L-alaninate (**12d**)

Prepared according to the same procedure as **12g** using benzyloxycarbonyl-glycine (0.50 g, 2.4 mmol, 1 eq), methyl *L*-alaninate HCl (0.33 g, 2.4 mmol, 1 eq), isobutyl chloroformate (0.49 g, 3.6 mmol, 1.5 eq) and N-methylmorpholine (0.97 g, 9.6 mmol, 4 eq) to produce **12d** (0.37 g, 1.24 mmol, 52%). ^{1}H NMR (500 MHz, DMSO-d_6) δ 8.28 (d, *J* = 7.1 Hz, 1H), 7.41 (t, *J* = 6.2 Hz, 1H), 7.38 – 7.18 (m, 5H), 4.99 (s, 2H), 4.35 – 4.15 (m, 1H), 3.73 – 3.50 (m, 5H), 1.23 (d, *J* = 7.3 Hz, 3H); APCI-MS: *m/z* 295.0 [M+H]$^+$.

Methyl ((benzyloxy)carbonyl)-L-alanyl-L-alaninate (**12e**)

Prepared according to the same procedure as **12g** using benzyloxycarbonyl-*L*-alanine (0.54 g, 2.4 mmol, 1 eq), methyl *L*-alaninate HCl (0.33 g, 2.4 mmol, 1 eq), isobutyl chloroformate (0.49 g, 3.6 mmol, 1.5 eq) and N-methylmorpholine (0.97 g, 9.6 mmol, 4 eq) to produce **12e** (0.39 g, 1.26 mmol, 52%). ^{1}H NMR (500 MHz, DMSO-d_6) δ 8.26 (d, *J* = 7.1 Hz, 1H), 7.40 (d, *J* = 7.8 Hz, 1H), 7.37 – 7.18 (m, 5H), 4.97 (d, *J* = 4.0 Hz, 2H), 4.31 – 4.11 (m, 1H), 4.10 – 3.93 (m, 1H), 3.57 (s, 3H), 1.24 (d, *J* = 7.3 Hz, 3H), 1.16 (d, *J* = 7.2 Hz, 3H); APCI-MS: *m/z* 309.0 [M+H]$^+$.

Methyl ((benzyloxy)carbonyl)-L-leucyl-L-alaninate (**12f**)

Prepared according to the same procedure as **12g** using benzyloxycarbonyl-*L*-leucine (0.64 g, 2.4 mmol, 1 eq), methyl *L*-alaninate HCl (0.33 g, 2.4 mmol, 1 eq), isobutyl chloroformate (0.49 g, 3.6 mmol, 1.5 eq) and N-methylmorpholine (0.97 g, 9.6 mmol, 4 eq) to produce **12f** (0.62 g, 1.76 mmol, 73%). ^{1}H NMR (500 MHz, DMSO-d_6) δ 8.32 (d, *J* = 6.9 Hz, 1H), 7.44 – 7.21 (m, 6H), 4.97 (s, 2H), 4.21 (p, *J* = 7.1 Hz, 1H), 4.10 – 3.94 (m, 1H), 3.56 (s, 3H), 1.69 – 1.53 (m, 1H), 1.50 – 1.31 (m, 2H), 1.24 (d, *J* = 7.3 Hz, 3H), 0.83 (dd, *J* = 9.8, 6.6 Hz, 6H); APCI-MS: *m/z* 351.2 $[M+H]^+$.

Methyl ((benzyloxy)carbonyl)-L-(O-tert-butyl)seryl-L-alaninate (**12h**)

Prepared according to the same procedure as **12g** using benzyloxycarbonyl-*L*-(O-tert-butyl)serine (0.71 g, 2.4 mmol, 1 eq), methyl *L*-alaninate HCl (0.33 g, 2.4 mmol, 1 eq), isobutyl chloroformate (0.49 g, 3.6 mmol, 1.5 eq) and N-methylmorpholine (0.97 g, 9.6 mmol, 4 eq) to produce **12h** (0.61 g, 1.60 mmol, 67%). ^{1}H NMR (500 MHz, DMSO-d_6) δ 8.29 (d, *J* = 7.2 Hz, 1H), 7.42 – 7.11 (m, 6H), 4.99 (d, *J* = 5.2 Hz, 2H), 4.33 – 4.18 (m, 1H), 4.18 – 3.97 (m, 1H), 3.57 (s, 3H), 3.52 – 3.42 (m, 1H), 1.24 (d, *J* = 7.3 Hz, 3H), 1.07 (s, 9H); APCI-MS: *m/z* 381.1 $[M+H]^+$.

Methyl ((benzyloxy)carbonyl)-L-(O-tert-butyl)threonyl-L-alaninate (**12i**)

Prepared according to the same procedure as **12g** using benzyloxycarbonyl-*L*-(O-tert-butyl)threonine (0.74 g, 2.4 mmol, 1 eq), methyl *L*-alaninate HCl (0.33 g, 2.4 mmol, 1 eq), isobutyl chloroformate (0.49 g, 3.6 mmol, 1.5 eq) and N-methylmorpholine (0.97 g, 9.6 mmol, 4 eq) to produce **12i** (0.66 g, 1.67 mmol, 69%). ^{1}H NMR (500 MHz, Chloroform-*d*) δ 8.16 (d, *J* = 6.9 Hz, 1H), 7.40 – 7.24 (m, 5H), 6.80 (d, *J* = 9.5 Hz, 1H), 5.01 (d, *J* = 2.6 Hz, 2H), 4.24 (p, *J* = 7.2 Hz, 1H), 4.03 – 3.88 (m, 1H), 3.87 – 3.75 (m, 1H), 3.58 (s, 3H), 1.25 (d, *J* = 7.3 Hz, 3H), 1.09 – 0.96 (m, 13H); APCI-MS: *m/z* 395.1 $[M+H]^+$.

Methyl ((benzyloxy)carbonyl)-L-(N4-trityl)asparaginyl-L-alaninate (**12j**)

Prepared according to the same procedure as **12g** using benzyloxycarbonyl-*L*-(N4-trityl)asparagine (1.2 g, 2.4 mmol, 1 eq), methyl *L*-alaninate HCl (0.33 g, 2.4 mmol, 1 eq), isobutyl chloroformate (0.49 g, 3.6 mmol, 1.5 eq) and N-methylmorpholine (0.97 g, 9.6 mmol, 4 eq) to produce **12j** (0.46 g, 0.77 mmol, 32%). ^{1}H NMR (500 MHz, Chloroform-*d*) δ 8.49 (s, 1H), 8.32 (d, *J* = 6.9 Hz, 1H), 7.52 (d, *J* = 8.4 Hz, 1H), 7.43 – 6.91 (m, 21H), 5.01 (d, *J* = 3.2 Hz, 2H), 4.38 – 4.25 (m, 1H), 4.25 – 4.12 (m, 1H), 3.56 (s, 3H), 2.70 – 2.53 (m, 1H), 1.00 (d, *J* = 6.1 Hz, 3H).

Methyl ((benzyloxy)carbonyl)-L-(O-tert-butyl)aspartyl-L-alaninate (**12k**)

Prepared according to the same procedure as **12g** using (S)-2-(((benzyloxy)carbonyl)amino)-4-(tert-butoxy)-4-oxobutanoic acid (0.78 g, 2.4 mmol, 1 eq), methyl *L*-alaninate HCl (0.33 g, 2.4 mmol, 1 eq), isobutyl chloroformate (0.49 g, 3.6 mmol, 1.5 eq) and N-methylmorpholine (0.97 g, 9.6 mmol, 4 eq) to produce **12k** (0.47 g, 1.1 mmol, 48%). ^{1}H NMR (500 MHz, DMSO-d_6) δ 8.34 (d, *J* = 7.1 Hz, 1H), 7.52 (d, *J* = 8.4 Hz, 1H), 7.30 (q, *J* = 12.4, 9.4 Hz, 5H), 4.99 (q, *J* = 12.6 Hz, 2H), 4.45 – 4.27 (m, 1H), 4.20 (p, *J* = 7.1 Hz, 1H), 3.57 (s, 3H), 2.58 (dd, *J* = 16.4, 4.4 Hz, 1H), 2.42 – 2.33 (m, 2H), 1.48 – 1.05 (m, 12H); APCI-MS: *m/z* 409.1 $[M+H]^+$.

Methyl ((benzyloxy)carbonyl)-L-valyl-D-alaninate (**12l**)

Prepared according to the same procedure as **12g** using benzyloxycarbonyl-*L*-valine (0.60 g, 2.4 mmol, 1 eq), methyl *D*-alaninate HCl (0.33 g, 2.4 mmol, 1 eq), isobutyl chloroformate (0.49 g, 3.6 mmol, 1.5 eq) and N-methylmorpholine (0.97 g, 9.6 mmol, 4 eq) to produce **12l** (0.32 g, 0.96 mmol, 40%). ^{1}H NMR (500 MHz, DMSO-d_6) δ 8.31 (d, *J* = 7.1 Hz, 1H), 7.41 – 7.16 (m, 6H), 5.00 (d, *J* = 1.4 Hz, 2H), 4.23 (p, *J* = 7.2 Hz, 1H), 3.87 (dd, *J* = 9.2, 6.9 Hz, 1H), 3.58 (s, 3H), 1.89 (h, *J* = 6.8 Hz, 1H), 1.22 (d, *J* = 7.3 Hz, 3H), 0.80 (t, *J* = 6.9 Hz, 6H); APCI-MS: *m/z* 337.2 [M+H]$^+$.

Methyl ((benzyloxy)carbonyl)-L-valyl-L-leucinate (**12m**)

Prepared according to the same procedure as **12g** using benzyloxycarbonyl-*L*-valine (0.60 g, 2.4 mmol, 1 eq), methyl *L*-leucinate HCl (0.44 g, 2.4 mmol, 1 eq), isobutyl chloroformate (0.49 g, 3.6 mmol, 1.5 eq) and N-methylmorpholine (0.97 g, 9.6 mmol, 4 eq) to produce **12m** (0.63 g, 1.7 mmol, 69%). ^{1}H NMR (500 MHz, DMSO-d_6) δ 8.25 (d, *J* = 7.4 Hz, 1H), 7.47 – 7.15 (m, 6H), 5.13 – 4.94 (m, 2H), 4.37 – 4.20 (m, 1H), 3.99 – 3.82 (m, 1H), 3.61 (s, 3H), 2.01 – 1.91 (m, 1H), 1.75 – 1.42 (m, 3H), 0.88 (dt, *J* = 24.4, 6.5 Hz, 12H); APCI-MS: *m/z* 379.2 [M+H]$^+$.

Methyl ((benzyloxy)carbonyl)-L-valyl-L-phenylalininate (**12n**)

Prepared according to the same procedure as **12g** using benzyloxycarbonyl-*L*-valine (0.60 g, 2.4 mmol, 1 eq), methyl *L*-phenylalininate HCl (0.52 g, 2.4 mmol, 1 eq), isobutyl chloroformate (0.49 g, 3.6 mmol, 1.5 eq) and N-methylmorpholine (0.97 g, 9.6 mmol, 4 eq) to produce **12n** (0.50 g, 1.2 mmol, 51%). ^{1}H NMR (500 MHz, DMSO-d_6) δ 8.36 (d, *J* = 7.4 Hz, 1H), 7.58 – 7.05 (m, 11H), 5.04 (s, 2H), 4.49 (dt, *J* = 8.9, 7.1 Hz, 1H), 3.89 (dd, *J* = 9.1, 7.1 Hz, 1H), 3.57 (s, 3H), 3.10 – 2.82 (m, 2H), 1.92 (h, *J* = 6.9 Hz, 1H), 0.82 (t, *J* = 6.8 Hz, 6H); APCI-MS: *m/z* 413.2 [M+H]$^+$.

Methyl ((benzyloxy)carbonyl)-L-valyl-L-(O-tert-butyl)serinate (**12o**)

Prepared according to the same procedure as **12g** using benzyloxycarbonyl-*L*-valine (0.60 g, 2.4 mmol, 1 eq), methyl *L*-(O-tert-butyl)serinate HCl (0.51 g, 2.4 mmol, 1 eq), isobutyl chloroformate (0.49 g, 3.6 mmol, 1.5 eq) and N-methylmorpholine (0.97 g, 9.6 mmol, 4 eq) to produce **12o** (0.83 g, 2.0 mmol, 85%). ^{1}H NMR (500 MHz, DMSO-d_6) δ 8.12 (d, *J* = 7.7 Hz, 1H), 7.43 – 7.25 (m, 6H), 5.05 (s, 2H), 4.45 (dt, *J* = 7.6, 4.8 Hz, 1H), 4.00 (dd, *J* = 9.1, 6.8 Hz, 1H), 3.63 (s, 3H), 3.52 (dd, *J* = 9.3, 4.6 Hz, 1H), 2.00 – 1.94 (m, 1H), 1.12 (s, 9H), 0.92 – 0.83 (m, 7H); APCI-MS: *m/z* 409.3 [M+H]$^+$.

Methyl ((benzyloxy)carbonyl)-L-valyl-L-(O-tert-butyl)threoninate (**12p**)

Prepared according to the same procedure as **12g** using benzyloxycarbonyl-*L*-valine (0.60 g, 2.4 mmol, 1 eq), methyl *L*-(O-tert-butyl)threoninate HCl (0.54 g, 2.4 mmol, 1 eq), isobutyl chloroformate (0.49 g, 3.6 mmol, 1.5 eq) and N-methylmorpholine (0.97 g, 9.6 mmol, 4 eq) to produce **12p** (0.81 g, 1.9 mmol, 80%). ^{1}H NMR (500 MHz, DMSO-d_6) δ 7.73 (d, *J* = 8.7 Hz, 1H), 7.46 – 7.25 (m, 5H), 5.14 – 4.89 (m, 2H), 4.40 (dd, *J* = 8.7, 2.8 Hz, 1H), 4.19 – 4.12 (m, 1H), 4.12 – 3.97 (m, 1H), 3.63 (s, 3H), 2.07 – 1.91 (m, 1H), 1.17 – 1.01 (m, 12H), 0.92 – 0.79 (m, 7H); APCI-MS: *m/z* 423.3 [M+H]$^+$.

Methyl ((benzyloxy)carbonyl)-L-valyl-L-(N4-trityl)asparaginate (**12q**)

Prepared according to the same procedure as **12g** using benzyloxycarbonyl-*L*-valine (0.60 g, 2.4 mmol, 1 eq), methyl *L*-(N4-trityl)asparaginate HCl (1.0 g, 2.4 mmol, 1 eq), isobutyl chloroformate (0.49 g, 3.6 mmol, 1.5 eq) and N-methylmorpholine (0.97 g, 9.6 mmol, 4 eq) to produce **12q** (0.38 g, 0.60 mmol, 25%). ^{1}H NMR (500 MHz, DMSO-d_6) δ 8.74 (s, 1H), 8.33 (d, *J* = 7.6 Hz, 1H), 7.50 – 7.12 (m, 19H), 5.07 – 5.03 (m, 2H), 4.66 – 4.47 (m, 1H), 3.99 – 3.84 (m, 1H), 3.58 (s, 3H), 2.79 (dd, *J* = 15.6, 6.2 Hz, 1H), 2.74 – 2.60 (m, 1H), 0.98 – 0.76 (m, 9H); APCI-MS: *m/z* 622.3 $[M+H]^+$.

Methyl ((benzyloxy)carbonyl)-L-valyl-L-(O-tert-butyl)aspartate (**12r**)

Prepared according to the same procedure as **12g** using benzyloxycarbonyl-*L*-valine (0.60 g, 2.4 mmol, 1 eq), methyl *L*-(O-tert-butyl)aspartate HCl (0.58 g, 2.4 mmol, 1 eq), isobutyl chloroformate (0.49 g, 3.6 mmol, 1.5 eq) and N-methylmorpholine (0.97 g, 9.6 mmol, 4 eq) to produce **12r** (0.52 g, 1.2 mmol, 49%). ^{1}H NMR (500 MHz, DMSO-d_6) δ 8.37 (d, *J* = 7.8 Hz, 1H), 7.47 – 7.19 (m, 6H), 5.04 (s, 2H), 4.63 (q, *J* = 7.1 Hz, 1H), 3.90 (dd, *J* = 9.0, 6.9 Hz, 1H), 3.62 (s, 3H), 2.72 (dd, *J* = 16.2, 5.9 Hz, 1H), 2.60 (dd, *J* = 16.3, 7.5 Hz, 1H), 2.06 – 1.85 (m, 1H), 1.39 (s, 9H), 0.87 (dd, *J* = 15.2, 6.8 Hz, 6H); APCI-MS: *m/z* 437.2 $[M+H]^+$.

Methyl ((benzyloxy)carbonyl)-L-valyl-L-(O-tert-butyl)glutamate (**12s**)

Prepared according to the same procedure as **12g** using benzyloxycarbonyl-*L*-valine (0.60 g, 2.4 mmol, 1 eq), methyl *L*-(O-tert-butyl)glutamate HCl (0.61 g, 2.4 mmol, 1 eq), isobutyl chloroformate (0.49 g, 3.6 mmol, 1.5 eq) and N-methylmorpholine (0.97 g, 9.6 mmol, 4 eq) to produce **12s** (0.36 g, 0.79 mmol, 33%). ^{1}H NMR (500 MHz, DMSO-d_6) δ 8.30 (d, *J* = 7.3 Hz, 1H), 7.45 – 7.16 (m, 6H), 5.05 (s, 2H), 4.38 – 4.20 (m, 1H), 3.99 – 3.82 (m, 1H), 3.62 (s, 3H), 2.31 (t, *J* = 7.5 Hz, 2H), 2.05 – 1.85 (m, 2H), 1.85 – 1.73 (m, 1H), 1.40 (s, 9H), 0.88 (dd, *J* = 17.9, 6.7 Hz, 6H); APCI-MS: *m/z* 451.3 $[M+H]^+$.

Methyl ((4-ethynylbenzyloxy)carbonyl)-L-valyl-L-alaninate (**12t**)

To a stirring solution of **38a** (0.32 g, 1.08 mmol, 1 eq) in DCM was added 4.0 M HCl in 1,4-dioxane (3 mL, 12.0 mmol, 11 eq). This was allowed to stir at 25 °C for 30 minutes before concentrating *in vacuo* and triturating with ether to remove excess HCl. The residue dissolved in THF (4 mL) and TEA (0.36 mL, 2.6 mmol, 2.4 eq) was added. To this was added **39** (0.21 g, 1.08 mmol, 1.01 eq) dissolved in THF dropwise at 0 °C and allowed to stir overnight at room temperature. The reaction was mixture was then diluted with EtOAc (25 mL), and washed with water (50 mL), 1 N HCl (50 mL), saturated sodium bicarbonate, and brine before drying over sodium sulfate, filtering, concentrating *in vacuo*, and triturating with hexanes to yield **12t** (0.092 g, 0.25 mmol, 24%). ^{1}H NMR (500 MHz, Chloroform-*d*) δ 7.48 (d, *J* = 8.1 Hz, 2H), 7.31 (d, *J* = 8.0 Hz, 2H), 6.26 (d, *J* = 7.4 Hz, 1H), 5.36 (d, *J* = 8.9 Hz, 1H), 5.10 (s, 2H), 4.59 (q, *J* = 7.1 Hz, 1H), 4.10 – 3.91 (m, 1H), 3.76 (s, 3H), 3.08 (s, 1H), 2.12 (q, *J* = 6.7 Hz, 1H), 1.42 (d, *J* = 7.2 Hz, 3H), 0.96 (dd, *J* = 21.5, 6.8 Hz, 5H); APCI-MS: *m/z* 361.4 $[M+H]^+$.

Methyl ((4-ethynylbenzyloxy)carbonyl)-L-valyl-glycinate (**12u**)

Prepared according to the procedure for **12t** using **38b** (0.10 g, 0.36 mmol, 1 eq), 4.0 M HCl in 1,4-dioxane (1 mL, 4.0 mmol, 11 eq), TEA (0.11 mL, 0.79 mmol, 2.2 eq) and **39** (0.077 g, 0.39 mmol, 1.1 eq) to yield **12u** (0.01 g, 0.03 mmol, 8%). ^{1}H NMR (500 MHz, Chloroform-*d*) δ 7.55 – 7.40 (m, 2H), 7.31 (d, *J* = 7.9

Hz, 2H), 6.40 – 6.25 (m, 1H), 5.34 (d, *J* = 8.3 Hz, 1H), 5.10 (s, 2H), 4.18 – 3.97 (m, 3H), 3.76 (s, 3H), 3.08 (s, 1H), 2.25 – 2.07 (m, 1H), 0.97 (dd, *J* = 21.6, 6.8 Hz, 6H); APCI-MS: *m/z* 345.0 $[M-H]^-$.

Methyl ((4-ethynylbenzyloxy)carbonyl)-L-phenylalaninyl-L-alaninate (**12v**)

Prepared according to the procedure for **12t** using **38c** (0.60 g, 1.7 mmol, 1 eq), 4.0 M HCl in 1,4-dioxane (5 mL, 19.0 mmol, 11 eq), TEA (0.37 g, 3.7 mmol, 2.2 eq) and **39** (0.36 g, 1.9 mmol, 1.1 eq) to yield **12v** (0.16 g, 0.40 mmol, 23%). ^{1}H NMR (500 MHz, DMSO-d_6) δ 8.49 (d, *J* = 7.1 Hz, 1H), 7.54 (d, *J* = 8.8 Hz, 1H), 7.46 – 7.04 (m, 9H), 4.90 (s, 2H), 4.34 – 4.19 (m, 2H), 4.16 (s, 1H), 3.58 (s, 3H), 2.96 (dd, *J* = 13.9, 3.7 Hz, 1H), 2.67 (dd, *J* = 13.8, 11.0 Hz, 1H), 1.28 (d, *J* = 7.3 Hz, 3H); APCI-MS: *m/z* 409.4 $[M+H]^+$.

General procedure for methylester deprotection to yield **13a-v.**

The dipeptide **12a-v** (1.0 eq) was dissolved in THF:H_2O (4:1, 10 mL) and LiOH (1.2 equiv) was added at 0 °C. The mixture was stirred at 0 °C for 3 hours before acidifying with 1 M HCl (2 mL) to < pH 3. The aqueous phase was extracted with EtOAc (3 X 5 mL), dried over sodium sulfate, filtered, and concentrated to yield **13a-v**, which were confirmed by MS then carried directly into the final step.

Cbz-L-Val-L-Ala-L-Glu(OMe)-fluoromethylketone (**1**)

Prepared according to the general procedure for **21** using **8a** (0.060 g, 0.22 mmol, 1.0 equiv), 4.0 M HCl in 1,4-dioxane (3.0 mL, 12.0 mmol, excess), **13a** (0.056 g, 0.17 mmol, 0.8 eq), isobutyl chloroformate (0.044 mL, 0.32 mmol, 1.5 eq) and *N*-methylmorpholine (0.095 mL, 0.86 mmol, 4.0 eq) to yield **1** as a white solid (0.045 g, 0.092 mmol, 43%). ^{1}H NMR (800 MHz, DMSO-d_6) δ 8.33 (d, *J* = 7.4 Hz, 1H), 8.10 (d, *J* = 6.7 Hz, 1H), 7.40 – 7.26 (m, 6H), 5.15 (dq, *J* = 50.5, 46.7, 16.6 Hz, 2H), 5.02 (q, *J* = 12.6 Hz, 2H), 4.31 – 4.26 (m, 1H), 4.23 (p, *J* = 7.0 Hz, 1H), 3.87 (dd, *J* = 8.6, 6.7 Hz, 1H), 3.57 (s, 3H), 2.40 – 2.25 (m, 2H), 2.07 – 2.00 (m, 1H), 1.95 (h, *J* = 6.8 Hz, 1H), 1.78 – 1.69 (m, 1H), 1.22 (d, *J* = 7.1 Hz, 3H), 0.84 (dd, *J* = 31.9, 6.8 Hz, 6H). ^{13}C NMR (201 MHz, DMSO-d_6) δ 203.7 (d, *J* = 14 Hz), 173.2, 173.1, 171.4, 156.6, 137.4, 128.7, 128.2, 128.0, 84.3 (d, *J* = 178 Hz), 65.8, 60.3, 54.7, 51.7, 48.5, 30.7, 29.7, 24.6, 19.5, 18.4, 18.0. ESI-MS: *m/z* 482.5 $[M+H]^+$; HPLC t_R =11.434 min (Column B); HPLC purity: 96%.

Cbz-L-Val-L-Ala-L-Glu(OMe)-chloromethylketone (**14**)

Prepared according to the general procedure for **21** using **11** (0.050 g, 0.17 mmol, 1.0 equiv), 4.0 M HCl in 1,4-dioxane (1.0 mL, 4.0 mmol, excess), **13a** (0.044 g, 0.14 mmol, 0.8 eq), isobutyl chloroformate (0.034 mL, 0.26 mmol, 1.5 eq) and *N*-methylmorpholine (0.075 mL, 0.68 mmol, 4.0 eq) to yield **14** as a white solid (0.022 g, 0.044 mmol, 26%). ^{1}H NMR (800 MHz, DMSO-d_6) δ 8.41 (d, *J* = 7.4 Hz, 1H), 8.11 (d, *J* = 6.7 Hz, 1H), 7.44 – 7.21 (m, 6H), 5.02 (q, *J* = 12.6 Hz, 2H), 4.54 (s, 2H), 4.33 – 4.26 (m, 1H), 4.23 (p, *J* = 7.0 Hz, 1H), 3.87 (dd, *J* = 8.6, 6.7 Hz, 1H), 3.57 (s, 3H), 2.38 – 2.27 (m, 2H), 2.08 – 2.01 (m, 1H), 1.97 – 1.92 (m, 1H), 1.79 – 1.70 (m, 1H), 1.23 (d, *J* = 7.1 Hz, 3H), 0.84 (dd, *J* = 30.4, 6.8 Hz, 6H). ^{13}C NMR (201 MHz, DMSO) δ 200.7, 173.2, 173.1, 171.5, 156.6, 137.4, 128.7, 128.2, 128.0, 65.8, 60.3, 56.1, 51.8, 48.6, 48.0, 40.4, 40.3, 30.7, 29.8, 24.9, 19.5, 18.5, 18.0; ESI-MS: *m/z* 498.3 $[M+H]^+$; HPLC t_R = 11.704 min (Column B); HPLC Purity: 97.6%.

Cbz-L-Val-Gly-L-Glu(OMe)-fluoromethylketone (**15**)

Prepared according to the general procedure for **21** using **8a** (0.050 g, 0.18 mmol, 1.0 equiv), 4.0 M HCl in 1,4-dioxane (2.0 mL, 8.0 mmol, excess), **13b** (0.044 g, 0.14 mmol, 0.8 eq), isobutyl chloroformate (0.036 mL, 0.27 mmol, 1.5 eq) and *N*-methylmorpholine (0.079 mL, 0.72 mmol, 4.0 eq) to yield **15** as a white solid (0.030 g, 0.065 mmol, 36%). ^{1}H NMR (500 MHz, Chloroform-*d*) δ 7.37 – 7.29 (m, 5H), 6.95 – 6.73 (m, 1H), 5.47 (d, *J* = 7.4 Hz, 1H), 5.20 – 4.88 (m, 4H), 4.86 – 4.72 (m, 1H), 4.18 – 3.82 (m, 3H), 3.70 – 3.55 (m, 3H), 2.52 – 2.29 (m, 2H), 2.31 – 2.19 (m, 1H), 2.18 – 2.05 (m, 1H), 2.02 – 1.84 (m, 1H), 1.74 (s, 1H), 0.97 (dd, *J* = 14.7, 6.9 Hz, 6H). ^{13}C NMR (126 MHz, $CDCl_3$) δ 203.8 (d, *J* = 14 Hz), 173.19, 171.95, 168.96, 156.67, 135.80, 128.47, 128.21, 128.10, 127.97, 84.0 (d, *J* = 181 Hz), 67.20, 61.13, 60.97, 54.46, 51.83, 42.84, 30.37, 29.50, 25.09, 19.16, 17.93. ESI-MS: *m/z* 468.1 $[M+H]^+$; HPLC t_R = 11.382 min (Column B); HPLC Purity: 97.8%.

Cbz-L-Val-Gly-L-Glu-fluoromethylketone (**16**)

Prepared according to the general procedure for **21** using **8b** (0.20 g, 0.62 mmol, 1.0 equiv), 4.0 M HCl in 1,4-dioxane (2.0 mL, 8.0 mmol, excess), **13b** (0.15 g, 0.50 mmol, 0.8 eq), isobutyl chloroformate (0.12 mL, 0.94 mmol, 1.5 eq) and *N*-methylmorpholine (0.28 mL, 2.5 mmol, 4.0 eq). The resulting residue was treated with a 4 mL solution of 95:2.5:2.5 TFA:Triisopropyl silane:water for 1 hour before evaporating the solvent and triturating with ether before purifying on HPLC (5 → 95 % ACN:H_2O over 30 minutes) to yield **16** as a white solid (0.013 g, 0.028 mmol, 4.5%). ^{1}H NMR (500 MHz, Chloroform-*d*) δ 7.64 (s, 1H), 7.52 (s, 1H), 7.37 – 7.27 (m, 5H), 5.86 (d, *J* = 7.6 Hz, 1H), 5.25 – 4.86 (m, 4H), 4.78 (s, 1H), 4.16 – 3.98 (m, 2H), 3.98 – 3.80 (m, 1H), 2.38 (s, 2H), 2.22 – 2.14 (m, 1H), 2.12 – 2.04 (m, 1H), 2.00 (s, 1H), 1.94 – 1.82 (m, 1H), 0.93 (dd, *J* = 16.7, 6.7 Hz, 6H). ^{13}C NMR (126 MHz, $CDCl_3$) δ 203.8 (d, *J* = 21 Hz), 175.86, 173.02, 170.01, 157.4, 135.64, 128.47, 128.24, 127.91, 83.9 (d, *J* = 182 Hz), 67.37, 60.82, 54.73, 42.82, 30.50, 29.44, 24.68, 22.54, 19.10, 17.77, 14.01, 1.79. ESI-MS: *m/z* 454.2 $[M+H]^+$; HPLC t_R = 10 .679 min (Column B); HPLC Purity: 98.9%.

Cbz-D-Val-L-Ala-L-Glu(OMe)-fluoromethylketone (**17**)

Prepared according to the general procedure for **21** using **8a** (0.050 g, 0.18 mmol, 1.0 equiv), 4.0 M HCl in 1,4-dioxane (1.0 mL, 4.0 mmol, excess), **13c** (0.047 g, 0.14 mmol, 0.8 eq), isobutyl chloroformate (0.036 mL, 0.27 mmol, 1.5 eq) and *N*-methylmorpholine (0.079 mL, 0.72 mmol, 4.0 eq) to yield **17** as a white solid (0.032 g, 0.067 mmol, 37%). ^{1}H NMR (800 MHz, DMSO-d_6) δ 8.31 (d, *J* = 7.1 Hz, 1H), 8.12 (d, *J* = 7.7 Hz, 1H), 7.45 (d, *J* = 7.7 Hz, 1H), 7.38 – 7.29 (m, 5H), 5.19 – 5.03 (m, 2H), 5.04 – 4.92 (m, 2H), 4.36 – 4.30 (m, 1H), 4.24 (p, *J* = 7.1 Hz, 1H), 3.79 (t, *J* = 7.6 Hz, 1H), 3.57 (s, 3H), 2.43 – 2.28 (m, 2H), 2.10 – 2.02 (m, 1H), 1.91 (h, *J* = 6.8 Hz, 1H), 1.81 – 1.74 (m, 1H), 1.24 (d, *J* = 7.2 Hz, 3H), 0.86 (dd, *J* = 13.2, 6.7 Hz, 6H). ^{13}C NMR (201 MHz, DMSO) δ 203.6 (d, *J* = 15 Hz), 173.1, 173.1, 171.9, 156.8, 137.2, 128.7, 128.2, 128.2, 128.1, 84.0 (d, *J* = 181 Hz), 65.9, 61.0, 54.6, 51.8, 48.6, 30.3, 29.7, 24.5, 19.5, 19.0, 17.9. ESI-MS: *m/z* 482.3 $[M+H]^+$; HPLC t_R = 7.327 min (Column A); HPLC purity: 99.5%.

Cbz-Gly-L-Ala-L-Glu(OMe)-fluoromethylketone (**18**)

Prepared according to the general procedure for **21** using **8a** (0.050 g, 0.18 mmol, 1.0 equiv), 4.0 M HCl in 1,4-dioxane (1.0 mL, 4.0 mmol, excess), **13d** (0.040 g, 0.14 mmol, 0.8 eq), isobutyl chloroformate (0.036 mL, 0.27 mmol, 1.5 eq) and *N*-methylmorpholine (0.079 mL, 0.72 mmol, 4.0 eq) to yield **18** as a white solid (0.014 g, 0.032 mmol, 18%). ^{1}H NMR (800 MHz, DMSO-d_6) δ 8.25 (d, *J* = 7.5 Hz, 1H), 8.13 (d, *J* = 6.9 Hz, 1H), 7.51 – 7.24 (m, 6H), 5.21 – 5.04 (m, 2H), 5.02 (s, 2H), 4.36 – 4.25 (m, 1H), 4.27 – 4.18 (m, 1H), 3.64 (d, *J* = 6.0 Hz, 2H), 3.58 (s, 3H), 2.41 – 2.25 (m, 2H), 2.12 – 1.98 (m, 1H), 1.80 – 1.70 (m, 1H), 1.23 (d, *J* = 7.2 Hz, 3H). ^{13}C NMR (201 MHz, DMSO) δ 203.7 (d, *J* = 14 Hz), 173.3, 173.1, 169.4, 156.9, 137.4, 128.7, 128.2, 128.1, 84.1 (d, *J*

= 181 Hz), 65.9, 54.7, 51.8, 48.7, 43.8, 29.8, 24.5, 18.1. ESI-MS: *m/z* 440.0 $[M+H]^+$; HPLC t_R = 10.598 min (Column B); HPLC Purity: 96.5%.

Cbz-L-Ala-L-Ala-L-Glu(OMe)-fluoromethylketone (**19**)

Prepared according to the general procedure for **21** using **8a** (0.050 g, 0.18 mmol, 1.0 equiv), 4.0 M HCl in 1,4-dioxane (1.0 mL, 4.0 mmol, excess), **13e** (0.042 g, 0.14 mmol, 0.8 eq), isobutyl chloroformate (0.036 mL, 0.27 mmol, 1.5 eq) and *N*-methylmorpholine (0.079 mL, 0.72 mmol, 4.0 eq) to yield **19** as a white solid (0.014 g, 0.032 mmol, 18%). ^{1}H NMR (800 MHz, DMSO-d_6) δ 8.24 (d, *J* = 7.4 Hz, 1H), 8.06 (d, *J* = 6.9 Hz, 1H), 7.49 (d, *J* = 7.2 Hz, 1H), 7.39 – 7.33 (m, 4H), 7.31 (t, *J* = 7.0 Hz, 1H), 5.29 – 4.88 (m, 4H), 4.33 – 4.25 (m, 1H), 4.21 (p, *J* = 7.1 Hz, 1H), 4.04 (p, *J* = 7.2 Hz, 1H), 3.57 (s, 3H), 2.41 – 2.27 (m, 2H), 2.12 – 1.95 (m, 1H), 1.83 – 1.58 (m, 1H), 1.23 (d, *J* = 7.1 Hz, 3H), 1.19 (d, *J* = 7.2 Hz, 3H). ^{13}C NMR (201 MHz, DMSO) δ 203.7 (d, *J* = 14 Hz), 173.3, 173.1, 156.2, 137.4, 128.7, 128.2, 128.1, 84.1 (d, *J* = 181 Hz), 65.8, 54.7, 51.8, 50.4, 48.7, 29.7, 24.6, 18.3, 18.0. ESI-MS: *m/z* 454.0 $[M+H]^+$; HPLC t_R = 10.740 min (Column B); HPLC Purity: 95.3%.

Cbz-L-Leu-L-Ala-L-Glu(OMe)-fluoromethylketone (**20**)

Prepared according to the general procedure for **21** using **8a** (0.050 g, 0.18 mmol, 1.0 equiv), 4.0 M HCl in 1,4-dioxane (1.0 mL, 4.0 mmol, excess), **13f** (0.048 g, 0.14 mmol, 0.8 eq), isobutyl chloroformate (0.036 mL, 0.27 mmol, 1.5 eq) and *N*-methylmorpholine (0.079 mL, 0.72 mmol, 4.0 eq) to yield **20** as a white solid (0.032 g, 0.065 mmol, 36%). ^{1}H NMR (800 MHz, DMSO-d_6) δ 8.27 (d, *J* = 7.4 Hz, 1H), 8.09 (d, *J* = 6.8 Hz, 1H), 7.44 (d, *J* = 8.1 Hz, 1H), 7.40 – 7.24 (m, 5H), 5.25 – 4.91 (m, 4H), 4.33 – 4.25 (m, 1H), 4.21 (p, *J* = 7.0 Hz, 1H), 4.08 – 3.97 (m, 1H), 3.58 (s, 3H), 2.39 – 2.25 (m, 2H), 2.07 – 2.00 (m, 1H), 1.82 – 1.71 (m, 1H), 1.71 – 1.53 (m, 1H), 1.48 – 1.38 (m, 2H), 1.23 (d, *J* = 7.1 Hz, 3H), 0.86 (dd, *J* = 12.9, 6.6 Hz, 6H). ^{13}C NMR (201 MHz, DMSO) δ 203.6 (d, *J* = 15 Hz), 173.2, 173.1, 172.7, 156.4, 137.4, 128.7, 128.1, 128.0, 84.2 (d, *J* = 179 Hz), 65.7, 54.7, 53.3, 51.8, 48.6, 40.9, 29.7, 24.6, 24.5, 23.5, 21.8, 17.9. ESI-MS: *m*/z 496.1 $[M+H]^+$; HPLC t_R = 11.842 min (Column B); HPLC Purity: 99.4%.

Cbz-L-Ser-L-Ala-L-Glu(OMe)-fluoromethylketone (**22**)

Prepared according to the general procedure for **16** using **8a** (0.050 g, 0.18 mmol, 1.0 eq), 4.0 M HCl in 1,4-dioxane (2.0 mL, 8.0 mmol, excess), **13h** (0.053 g, 0.14 mmol, 0.8 eq), isobutyl chloroformate (0.036 mL, 0.27 mmol, 1.5 eq) and *N*-methylmorpholine (0.79 mL, 0.72 mmol, 4.0 eq) to yield **22** as a white solid (0.016 g, 0.034 mmol, 19%). ^{1}H NMR (800 MHz, DMSO-d_6) δ 8.23 (d, *J* = 6.9 Hz, 1H), 8.18 (d, *J* = 7.7 Hz, 1H), 7.40 – 7.27 (m, 6H), 5.22 – 4.97 (m, 4H), 4.33 – 4.26 (m, 1H), 4.23 (p, *J* = 7.1 Hz, 1H), 4.10 (q, *J* = 6.6 Hz, 1H), 3.58 (s, 6H), 2.38 – 2.21 (m, 2H), 2.12 – 1.94 (m, 1H), 1.79 – 1.64 (m, 1H), 1.25 (d, *J* = 7.2 Hz, 3H). ^{13}C NMR (201 MHz, DMSO) δ 203.6 (d, *J* = 14 Hz), 173.3, 173.1, 170.7, 156.4, 137.3, 128.7, 128.2, 128.1, 84.2 (d, *J* = 178 Hz), 65.9, 62.2, 57.2, 54.7, 51.8, 48.9, 29.7, 24.6, 17.9. ESI-MS: *m/z* 470.0 $[M+H]^+$; HPLC t_R = 10.354 min (Column B); HPLC Purity: 95.2%.

Cbz-L-Thr-L-Ala-L-Glu(OMe)-fluoromethylketone (**23**)

Prepared according to the general procedure for **16** using **8a** (0.050 g, 0.18 mmol, 1.0 eq), 4.0 M HCl in 1,4-dioxane (1.0 mL, 4.0 mmol, excess), **13i** (0.055 g, 0.14 mmol, 0.8 eq), isobutyl chloroformate (0.036 mL, 0.27 mmol, 1.5 eq) and *N*-methylmorpholine (0.79 mL, 0.72 mmol, 4.0 eq) to yield **23** as a white solid (0.010 g, 0.022 mmol, 12%). ^{1}H NMR (800 MHz, DMSO-d_6) δ 8.24 (d, *J* = 7.4 Hz, 1H), 8.10 (d, *J* = 6.8 Hz, 1H), 7.39 –

7.28 (m, 5H), 7.02 (d, *J* = 8.4 Hz, 1H), 5.27 – 4.95 (m, 4H), 4.32 – 4.18 (m, 2H), 3.98 (dd, *J* = 8.5, 4.7 Hz, 1H), 3.94 (q, *J* = 5.9 Hz, 1H), 3.58 (s, 4H), 2.40 – 2.25 (m, 2H), 2.07 – 1.97 (m, 1H), 1.78 – 1.66 (m, 1H), 1.24 (d, *J* = 7.2 Hz, 3H), 1.06 (d, *J* = 6.4 Hz, 3H). ^{13}C NMR (201 MHz, DMSO) δ 203.6 (d, *J* = 14 Hz), 173.2, 173.1, 170.4, 156.5, 137.3, 128.7, 128.2, 128.0, 84.2 (d, *J* = 180 Hz), 67.2, 65.9, 60.7, 54.7, 51.8, 48.7, 29.7, 24.6, 20.0, 18.0. ESI-MS: *m/z* 484.1 [M+H]$^+$; HPLC t_R = 10.583 min (Column B); HPLC Purity: 98.2%.

Cbz-L-Asn-L-Ala-L-Glu(OMe)-fluoromethylketone (**24**)

Prepared according to the general procedure for **16** using **8a** (0.050 g, 0.18 mmol, 1.0 eq), 4.0 M HCl in 1,4-dioxane (1.0 mL, 4.0 mmol, excess), **13j** (0.084 g, 0.14 mmol, 0.8 eq), isobutyl chloroformate (0.036 mL, 0.27 mmol, 1.5 eq) and *N*-methylmorpholine (0.79 mL, 0.72 mmol, 4.0 eq) to yield **24** as a white solid (0.009 g, 0.017 mmol, 10%). ^{1}H NMR (800 MHz, DMSO-*d*$_6$) δ 8.28 (d, *J* = 7.0 Hz, 2H), 7.49 (d, *J* = 7.9 Hz, 1H), 7.47 – 7.42 (m, 1H), 7.39 – 7.28 (m, 5H), 6.97 (s, 1H), 5.23 – 4.93 (m, 4H), 4.32 (q, *J* = 7.3 Hz, 1H), 4.30 – 4.23 (m, 1H), 4.15 (p, *J* = 7.1 Hz, 1H), 3.58 (s, 3H), 2.55 (dd, *J* = 15.3, 6.7 Hz, 1H), 2.45 – 2.39 (m, 1H), 2.39 – 2.26 (m, 2H), 2.11 – 1.94 (m, 1H), 1.84 – 1.70 (m, 1H), 1.24 (d, *J* = 7.3 Hz, 3H). ^{13}C NMR (201 MHz, DMSO) δ 203.6 (d, *J* = 14 Hz), 173.4, 173.1, 172.2, 137.1, 128.7, 128.1, 84.1 (d, *J* = 184 Hz), 65.9, 54.9, 51.8, 49.1, 37.7, 29.7, 24.5, 17.7. ESI-MS: *m/z* 496.9 [M+H]$^+$; HPLC t_R = 10.042 min (Column B); HPLC Purity: 95.3%.

Cbz-L-Asp-L-Ala-L-Glu(OMe)-fluoromethylketone (**25**)

Prepared according to the general procedure for **16** using **8a** (0.050 g, 0.18 mmol, 1.0 eq), 4.0 M HCl in 1,4-dioxane (1.0 mL, 4.0 mmol, excess), **13k** (0.057 g, 0.14 mmol, 0.8 eq), isobutyl chloroformate (0.036 mL, 0.27 mmol, 1.5 eq) and *N*-methylmorpholine (0.79 mL, 0.72 mmol, 4.0 eq) to yield **25** as a white solid (0.009 g, 0.017 mmol, 10%). ^{1}H NMR (800 MHz, DMSO-*d*$_6$) δ 8.21 (d, *J* = 7.5 Hz, 1H), 8.13 (d, *J* = 6.9 Hz, 1H), 7.61 (d, *J* = 7.9 Hz, 1H), 7.45 – 7.22 (m, 6H), 5.32 – 4.81 (m, 4H), 4.36 (td, *J* = 8.5, 4.5 Hz, 1H), 4.28 (ddd, *J* = 9.7, 7.5, 4.6 Hz, 1H), 4.18 (p, *J* = 7.1 Hz, 1H), 3.58 (s, 3H), 2.69 (dd, *J* = 16.7, 4.6 Hz, 1H), 2.41 – 2.22 (m, 2H), 2.12 – 1.96 (m, 1H), 1.82 – 1.66 (m, 1H), 1.23 (d, *J* = 7.3 Hz, 3H). ^{13}C NMR (201 MHz, DMSO) δ 203.6 (d, *J* = 14 Hz), 173.2, 173.1, 172.2, 171.3, 156.3, 137.3, 128.7, 128.2, 128.1, 84.2 (d, *J* = 182 Hz), 65.9, 54.8, 51.8, 51.6, 48.9, 36.5, 29.7, 25.9, 24.5, 17.9. ESI-MS: *m/z* 497.9 [M+H]$^+$; HPLC t_R = 10.415 min (Column B); HPLC Purity: 95.8%.

Cbz-L-Val-D-Ala-L-Glu(OMe)-fluoromethylketone (**26**)

Prepared according to the general procedure for **21** using **8a** (0.050 g, 0.18 mmol, 1.0 equiv), 4.0 M HCl in 1,4-dioxane (1.0 mL, 4.0 mmol, excess), **13l** (0.047 g, 0.14 mmol, 0.8 eq), isobutyl chloroformate (0.036 mL, 0.27 mmol, 1.5 eq) and *N*-methylmorpholine (0.079 mL, 0.72 mmol, 4.0 eq) to yield **26** as a white solid (0.028 g, 0.059 mmol, 33%). ^{1}H NMR (800 MHz, DMSO-*d*$_6$) δ 8.26 (d, *J* = 7.0 Hz, 1H), 8.18 (d, *J* = 7.7 Hz, 1H), 7.48 – 7.20 (m, 6H), 5.36 – 4.91 (m, 4H), 4.36 – 4.29 (m, 1H), 4.23 (p, *J* = 7.1 Hz, 1H), 3.82 (t, *J* = 7.7 Hz, 1H), 3.56 (s, 3H), 2.11 – 1.98 (m, 1H), 1.91 (h, *J* = 6.9 Hz, 1H), 1.84 – 1.69 (m, 1H), 1.22 (d, *J* = 7.1 Hz, 3H), 0.85 (t, *J* = 7.3 Hz, 7H). ^{13}C NMR (201 MHz, DMSO-*d*$_6$) δ 203.8 (d, *J* = 14 Hz), 173.2, 173.0, 171.7, 156.7, 137.3, 128.7, 128.2, 128.1, 84.6 (d, *J* = 179 Hz), 65.9, 60.8, 54.6, 51.8, 48.6, 30.4, 29.7, 24.7, 19.5, 18.9, 18.0. ESI-MS: *m/z* 482.1 [M+H]$^+$; HPLC t_R = 11.413 min (Column B); HPLC Purity: 97.9%.

Cbz-L-Val-L-Leu-L-Glu(OMe)-fluoromethylketone (**27**)

Prepared according to the general procedure for **21** using **8a** (0.050 g, 0.18 mmol, 1.0 equiv), 4.0 M HCl in 1,4-dioxane (1.0 mL, 4.0 mmol, excess), **13m** (0.053 g, 0.14 mmol, 0.8 eq), isobutyl chloroformate (0.036 mL, 0.27 mmol, 1.5 eq) and *N*-methylmorpholine (0.079 mL, 0.72 mmol, 4.0 eq) to yield **27** as a white solid (0.026 g, 0.049 mmol, 27%). ^{1}H NMR (800 MHz, DMSO-*d*$_6$) δ 8.38 (d, *J* = 7.4 Hz, 1H), 8.00 (d, *J* = 7.7 Hz,

1H), 7.38 – 7.27 (m, 6H), 5.21 – 4.97 (m, 4H), 4.35 – 4.23 (m, 2H), 3.90 – 3.82 (m, 1H), 3.57 (s, 3H), 2.39 – 2.27 (m, 2H), 2.07 – 2.00 (m, 1H), 1.95 (h, *J* = 6.8 Hz, 1H), 1.77 – 1.70 (m, 1H), 1.64 – 1.57 (m, 1H), 1.52 – 1.40 (m, 2H), 0.93 – 0.79 (m, 13H). ^{13}C NMR (201 MHz, DMSO-d_6) δ 203.5 (d, *J* = 13 Hz), 173.1, 172.9, 171.6, 156.6, 137.4, 128.7, 128.6, 128.2, 128.0, 127.4, 84.4 (d, *J* = 182 Hz), 65.8, 60.5, 54.6, 51.7, 51.3, 40.7, 30.6, 29.7, 24.6, 24.5, 23.3, 22.0, 19.5, 18.5. ESI-MS: *m/z* 524.3 [M+H]$^+$; HPLC t_R = 7.805 min (Column A); HPLC Purity: 96.1%.

Cbz-L-Val-L-Phe-L-Glu(OMe)-fluoromethylketone (**28**)

Prepared according to the general procedure for **21** using **8a** (0.050 g, 0.18 mmol, 1.0 equiv), 4.0 M HCl in 1,4-dioxane (1.0 mL, 4.0 mmol, excess), **13n** (0.057 g, 0.14 mmol, 0.8 eq), isobutyl chloroformate (0.036 mL, 0.27 mmol, 1.5 eq) and *N*-methylmorpholine (0.079 mL, 0.72 mmol, 4.0 eq) to yield **28** as a white solid (0.034 g, 0.061 mmol, 34%). ^{1}H NMR (800 MHz, DMSO-d_6) δ 8.42 (d, *J* = 7.7 Hz, 1H), 8.21 (d, *J* = 7.7 Hz, 1H), 7.40 – 7.14 (m, 12H), 5.08 – 4.95 (m, 2H), 4.94 – 4.70 (m, 2H), 4.52 (q, *J* = 7.7 Hz, 1H), 4.25 – 4.18 (m, 1H), 3.90 – 3.80 (m, 1H), 3.56 (s, 3H), 2.97 (dd, *J* = 13.7, 6.9 Hz, 1H), 2.86 (dd, *J* = 13.7, 8.3 Hz, 1H), 2.27 – 2.15 (m, 2H), 2.03 – 1.94 (m, 1H), 1.88 (h, *J* = 6.8 Hz, 1H), 1.72 – 1.59 (m, 1H), 0.77 (d, *J* = 6.8 Hz, 6H). ^{13}C NMR (201 MHz, DMSO-d_6) δ 203.3 (d, *J* = 14 Hz), 173.1, 171.8, 171.5, 156.5, 137.7, 137.4, 129.6, 128.7, 128.6, 128.5, 128.2, 128.1, 127.5, 126.8, 84.1 (d, *J* = 178 Hz), 65.8, 60.5, 54.6, 54.3, 51.7, 37.6, 30.7, 29.5, 24.6, 19.4, 18.4. ESI-MS: *m/z* 558.3 [M+H]$^+$; HPLC t_R = 7.844 min (Column A); HPLC Purity: 99.6%.

Cbz-L-Val-L-Ser-L-Glu(OMe)-fluoromethylketone (**29**)

Prepared according to the general procedure for **16** using **8a** (0.050 g, 0.18 mmol, 1.0 eq), 4.0 M HCl in 1,4-dioxane (1.0 mL, 4.0 mmol, excess), **13o** (0.057 g, 0.14 mmol, 0.8 eq), isobutyl chloroformate (0.036 mL, 0.27 mmol, 1.5 eq) and *N*-methylmorpholine (0.79 mL, 0.72 mmol, 4.0 eq) to yield **29** as a white solid (0.010 g, 0.019 mmol, 11%). ^{1}H NMR (800 MHz, DMSO-d_6) δ 8.37 (d, *J* = 7.6 Hz, 1H), 8.00 (d, *J* = 7.3 Hz, 1H), 7.41 – 7.25 (m, 6H), 5.29 – 4.94 (m, 5H), 4.38 – 4.17 (m, 2H), 3.91 (t, *J* = 7.7 Hz, 1H), 3.68 – 3.51 (m, 5H), 2.38 – 2.25 (m, 2H), 2.09 – 1.90 (m, 2H), 1.82 – 1.71 (m, 1H), 0.84 (dd, *J* = 28.6, 6.8 Hz, 7H). ^{13}C NMR (201 MHz, DMSO-d_6) δ 203.6 (d, *J* = 14 Hz), 173.2, 171.6, 171.2, 156.7, 137.4, 128.7, 128.2, 128.0, 120.0, 84.3 (d, *J* = 180 Hz), 65.8, 61.8, 60.5, 55.4, 54.9, 51.7, 30.7, 29.6, 24.5, 19.5, 18.4. ESI-MS: *m/z* 498.3 [M+H]$^+$; HPLC t_R = 7.002 min (Column A); HPLC Purity:95.7%.

Cbz-L-Val-L-Thr-L-Glu(OMe)-fluoromethylketone (**30**)

Prepared according to the general procedure for **16** using **8a** (0.050 g, 0.18 mmol, 1.0 eq), 4.0 M HCl in 1,4-dioxane (1.0 mL, 4.0 mmol, excess), **13p** (0.059 g, 0.14 mmol, 0.8 eq), isobutyl chloroformate (0.036 mL, 0.27 mmol, 1.5 eq) and *N*-methylmorpholine (0.79 mL, 0.72 mmol, 4.0 eq) to yield **30** as a white solid (0.007 g, 0.013 mmol, 7%). ^{1}H NMR (800 MHz, DMSO-d_6) δ 8.21 (d, *J* = 7.4 Hz, 1H), 7.72 (d, *J* = 8.1 Hz, 1H), 7.46 (d, *J* = 8.5 Hz, 1H), 7.41 – 7.28 (m, 5H), 5.26 – 5.08 (m, 2H), 5.04 (s, 2H), 4.35 – 4.27 (m, 2H), 4.23 – 4.15 (m, 2H), 4.02 – 3.92 (m, 2H), 3.56 (s, 3H), 2.40 – 2.25 (m, 2H), 2.08 – 1.94 (m, 2H), 1.81 – 1.68 (m, 1H), 1.05 (d, *J* = 6.3 Hz, 3H), 0.90 – 0.81 (m, 6H). ^{13}C NMR (201 MHz, DMSO-d_6) δ 203.6 (d, *J* = 14 Hz), 173.1, 171.8, 171.0, 158.6, 158.4, 158.3, 156.7, 137.4, 128.7, 128.2, 128.0, 117.3, 115.8, 84.4 (d, *J* = 180 Hz), 66.9, 65.8, 60.8, 58.6, 54.8, 51.7, 30.4, 29.6, 24.6, 20.3, 19.6, 18.5. ESI-MS: *m/z* 512.3 [M+H]$^+$; HPLC t_R = 7.154 min (Column A); HPLC Purity: 97.9%.

Cbz-L-Val-L-Asn-L-Glu(OMe)-fluoromethylketone (**31**)

Prepared according to the general procedure for **16** using **8a** (0.050 g, 0.18 mmol, 1.0 eq), 4.0 M HCl in 1,4-dioxane (1.0 mL, 4.0 mmol, excess), **13q** (0.088 g, 0.14 mmol, 0.8 eq), isobutyl chloroformate (0.036 mL, 0.27 mmol, 1.5 eq) and *N*-methylmorpholine (0.79 mL, 0.72 mmol, 4.0 eq) to yield **31** as a white solid (0.004 g, 0.008 mmol, 4%). ^{1}H NMR (800 MHz, DMSO-d_6) δ 8.24 (dd, *J* = 11.5, 7.8 Hz, 2H), 7.44 – 7.27 (m, 7H), 6.95 (s, 1H), 6.54 (s, 2H), 5.36 – 4.87 (m, 4H), 4.49 (q, *J* = 7.2 Hz, 1H), 4.24 (d, *J* = 10.6 Hz, 1H), 3.85 (t, *J* = 7.5 Hz, 1H), 3.56 (d, *J* = 1.9 Hz, 3H), 2.58 (dd, *J* = 15.7, 7.4 Hz, 1H), 2.45 (dd, *J* = 15.7, 6.6 Hz, 1H), 2.39 – 2.31 (m, 1H), 2.27 (dt, *J* = 16.6, 8.1 Hz, 1H), 2.01 (dq, *J* = 13.2, 6.6, 5.9 Hz, 1H), 1.94 (dt, *J* = 13.9, 7.0 Hz, 1H), 1.79 – 1.70 (m, 1H), 0.87 – 0.80 (m, 6H). ^{13}C NMR (201 MHz, DMSO-d_6) δ 203.6 (d, *J* = 14 Hz), 173.2, 171.8, 171.7, 171.5, 156.8, 137.3, 128.7, 128.2, 128.1, 84.4 (d, *J* = 179 Hz), 65.9, 60.5, 55.0, 51.7, 50.1, 36.9, 30.6, 29.5, 24.5, 19.4, 18.5. ESI-MS: *m/z* 525.3 [M+H]$^+$; HPLC t_R = 6.866 min (Column A); HPLC Purity: 98.2%.

Cbz-L-Val-L-Asp-L-Glu(OMe)-fluoromethylketone (**32**)

Prepared according to the general procedure for **16** using **8a** (0.050 g, 0.18 mmol, 1.0 eq), 4.0 M HCl in 1,4-dioxane (1.0 mL, 4.0 mmol, excess), **13r** (0.061 g, 0.14 mmol, 0.8 eq), isobutyl chloroformate (0.036 mL, 0.27 mmol, 1.5 eq) and *N*-methylmorpholine (0.79 mL, 0.72 mmol, 4.0 eq) to yield **32** as a white solid (0.014 g, 0.026 mmol, 15%). ^{1}H NMR (800 MHz, DMSO-d_6) δ 8.37 – 8.21 (m, 2H), 7.46 – 7.23 (m, 6H), 5.29 – 4.90 (m, 4H), 4.53 (d, *J* = 7.2 Hz, 1H), 4.28 (dt, *J* = 7.9, 4.0 Hz, 1H), 3.85 (t, *J* = 7.4 Hz, 2H), 3.56 (s, 3H), 2.73 (dd, *J* = 16.7, 6.5 Hz, 1H), 2.57 (dd, *J* = 16.7, 7.3 Hz, 1H), 2.39 – 2.32 (m, 1H), 2.32 – 2.23 (m, 1H), 2.07 – 1.86 (m, 2H), 1.82 – 1.66 (m, 1H), 0.84 (dd, *J* = 18.8, 6.7 Hz, 6H). ^{13}C NMR (201 MHz, DMSO-d_6) δ 203.5 (d, *J* = 14 Hz) 173.2, 172.2, 171.6, 171.4, 158.6, 158.4, 156.8, 137.3, 128.7, 128.2, 128.1, 127.6, 116.0, 84.4 (d, *J* = 182 Hz), 65.9, 60.6, 54.9, 51.7, 49.9, 36.0, 30.6, 29.5, 24.5, 19.4, 18.4. ESI-MS: *m/z* 526.3 [M+H]$^+$; HPLC t_R = 7.083 min (Column B); HPLC Purity: 98.6%.

Cbz-L-Val-L-Glu-L-Glu(OMe)-fluoromethylketone (**33**)

Prepared according to the general procedure for **16** using **8a** (0.050 g, 0.18 mmol, 1.0 eq), 4.0 M HCl in 1,4-dioxane (1.0 mL, 4.0 mmol, excess), **13s** (0.063 g, 0.14 mmol, 0.8 eq), isobutyl chloroformate (0.036 mL, 0.27 mmol, 1.5 eq) and *N*-methylmorpholine (0.79 mL, 0.72 mmol, 4.0 eq) to yield **33** as a white solid (0.007 g, 0.013 mmol, 7%). ^{1}H NMR (800 MHz, DMSO-d_6) δ 8.36 (d, *J* = 7.6 Hz, 1H), 8.07 (d, *J* = 7.5 Hz, 1H), 7.41 – 7.26 (m, 6H), 6.54 (s, 1H), 5.16 (dq, *J* = 47.2, 16.8 Hz, 2H), 5.02 (q, *J* = 12.7 Hz, 2H), 4.37 – 4.29 (m, 1H), 4.29 – 4.20 (m, 1H), 3.87 (t, *J* = 7.5 Hz, 1H), 3.62 – 3.54 (m, 3H), 2.41 – 2.11 (m, 4H), 2.09 – 1.66 (m, 5H), 0.84 (dd, *J* = 23.4, 6.7 Hz, 6H). ^{13}C NMR (201 MHz, DMSO-d_6) δ 203.6 (d, *J* = 14 Hz), 174.2, 173.1, 172.1, 171.7, 156.6, 137.4, 128.7, 128.2, 128.1, 84.5 (d, *J* = 178 Hz), 65.8, 60.5, 54.6, 52.3, 51.7, 30.6, 30.4, 29.7, 27.2, 24.6, 19.5, 18.5. ESI-MS: *m/z* 540.3 [M+H]$^+$; HPLC t_R = 7.074 min (Column B); HPLC Purity: 95.3%.

4-Ethynyl-Cbz-L-Val-L-Ala-L-Glu(OMe)-fluoromethylketone (**34**)

Prepared according to the general procedure for **21** using **8a** (0.040 g, 0.14 mmol, 1.0 eq), 4.0 M HCl in 1,4-dioxane (3.0 mL, 12.0 mmol, excess), **13t** (0.040 g, 0.12 mmol, 0.8 eq), isobutyl chloroformate (0.028 mL, 0.22 mmol, 1.5 eq) and *N*-methylmorpholine (0.063 mL, 0.58 mmol, 4.0 eq) to yield **34** as a white solid (0.039 g, 0.076 mmol, 53%). ^{1}H NMR (800 MHz, DMSO-d_6) δ 8.33 (d, *J* = 7.4 Hz, 1H), 8.10 (d, *J* = 6.7 Hz, 1H), 7.47 (d, *J* = 8.0 Hz, 2H), 7.40 – 7.31 (m, 3H), 5.14 (dq, 2H), 5.04 (q, 2H), 4.31 – 4.25 (m, 1H), 4.23 (p, *J* = 7.0 Hz, 1H), 4.18 (s, 1H), 3.87 (dd, *J* = 8.6, 6.6 Hz, 1H), 3.57 (s, 3H), 2.39 – 2.27 (m, 2H), 2.07 – 1.99 (m, 1H), 1.99 – 1.91 (m, 1H), 1.79 – 1.70 (m, 1H), 1.22 (d, *J* = 7.1 Hz, 3H), 0.84 (dd, *J* = 31.0, 6.8 Hz, 6H). ^{13}C NMR (201 MHz,

DMSO-d_6) δ 203.5 (d, *J* = 14 Hz), 173.2, 173.1, 132.1, 128.1, 84.4 (d, *J* = 178 Hz), 83.7, 81.3, 65.2, 60.3, 54.7, 51.7, 48.5, 40.4, 40.3, 30.7, 29.7, 24.6, 19.5, 18.4, 18.0. ESI-MS: *m/z* 506.2 $[M+H]^+$; HPLC t_R = 11.704 min (Column B); HPLC Purity: 97.9%.

4-Ethynyl-Cbz-L-Val-L-Gly-L-Glu(OMe)-fluoromethylketone (**35**)

Prepared according to the general procedure for **21** using **8a** (0.036 g, 0.13 mmol, 1.0 eq), 4.0 M HCl in 1,4-dioxane (2.0 mL, 8.0 mmol, excess), **13u** (0.034 g, 0.10 mmol, 0.8 eq), isobutyl chloroformate (0.025 mL, 0.19 mmol, 1.5 eq) and *N*-methylmorpholine (0.057 mL, 0.52 mmol, 4.0 eq) to yield **35** as a white solid (0.014 g, 0.029 mmol, 23%). ^{1}H NMR (500 MHz, Chloroform-*d*) δ 7.51 – 7.43 (m, 2H), 7.31 – 7.27 (m, 2H), 6.95 (t, *J* = 5.6 Hz, 1H), 5.55 (d, *J* = 7.7 Hz, 1H), 5.16 – 4.89 (m, 5H), 4.82 (q, 1H), 4.16 – 3.79 (m, 4H), 3.68 – 3.61 (m, 3H), 3.09 (s, 1H), 2.52 – 2.31 (m, 2H), 2.28 – 2.17 (m, 1H), 2.12 (q, *J* = 6.7 Hz, 1H), 1.92 (dq, *J* = 14.8, 7.5 Hz, 1H), 1.80 (s, 1H), 0.96 (dd, *J* = 12.3, 6.8 Hz, 6H). ^{13}C NMR (126 MHz, $CDCl_3$) δ 203.9 (d, *J* = 14 Hz), 173.2, 171.9, 168.9, 156.5, 136.6, 132.2, 127.7, 121.9, 84.0 (d, *J* = 181 Hz), 83.1, 77.6, 66.5, 60.9, 54.4, 51.8, 42.8, 30.5, 29.5, 25.1, 19.1, 18.9, 17.9. ESI-MS: *m/z* 492.2 $[M+H]^+$; HPLC t_R = 11.639 min (Column B); HPLC Purity: 96.5%.

4-Ethynyl-Cbz-L-Phe-L-Gly-L-Glu(OMe)-fluoromethylketone (**36**)

Prepared according to the general procedure for **21** using **8a** (0.150 g, 0.54 mmol, 1.0 eq), 4.0 M HCl in 1,4-dioxane (2.0 mL, 8.0 mmol, excess), **13v** (0.17 g, 0.43 mmol, 0.8 eq), isobutyl chloroformate (0.11 mL, 0.81 mmol, 1.5 eq) and *N*-methylmorpholine (0.24 mL, 2.2 mmol, 4.0 eq) to yield **36** as a white solid (0.017 g, 0.031 mmol, 6%). ^{1}H NMR (800 MHz, DMSO-d_6) δ 8.36 (d, *J* = 7.5 Hz, 1H), 8.30 (d, *J* = 6.8 Hz, 1H), 7.58 (d, *J* = 8.6 Hz, 1H), 7.43 (d, *J* = 7.9 Hz, 2H), 7.37 – 7.11 (m, 8H), 5.24 – 5.06 (m, 2H), 4.94 (s, 2H), 4.33 – 4.20 (m, 3H), 4.18 (s, 1H), 3.57 (s, 3H), 3.02 (dd, *J* = 14.0, 3.6 Hz, 1H), 2.71 (dd, *J* = 13.9, 11.0 Hz, 1H), 2.41 – 2.29 (m, 2H), 2.09 – 2.01 (m, 1H), 1.82 – 1.72 (m, 1H), 1.26 (d, *J* = 7.1 Hz, 3H). ^{13}C NMR (201 MHz, DMSO-d_6) δ 203.6 (d, *J* = 14 Hz), 203.6, 173.3, 173.1, 171.9, 156.2, 138.5, 138.4, 132.0, 131.9, 129.6, 128.4, 127.8, 127.3, 126.6, 121.3, 84.4 (d, *J* = 178 Hz), 83.7, 81.3, 65.0, 56.4, 54.7, 51.8, 48.8, 29.7, 24.6, 18.0. ESI-MS: *m/z* 554.3 $[M+H]^+$; HPLC t_R = 7.673 min (Column A); HPLC Purity: 95.5%.

4-Ethynyl-Cbz-L-Val-L-Ala-L-Glu(OMe)-fluoromethylketone (**37**)

Prepared according to the general procedure for **21** using **11** (0.043 g, 0.15 mmol, 1.0 eq), 4.0 M HCl in 1,4-dioxane (2.0 mL, 8.0 mmol, excess), **13t** (0.41 g, 0.12 mmol, 0.8 eq), isobutyl chloroformate (0.029 mL, 0.22 mmol, 1.5 eq) and *N*-methylmorpholine (0.064 mL, 0.59 mmol, 4.0 eq) to yield **37** as a white solid (0.025 g, 0.047 mmol, 32%). ^{1}H NMR (800 MHz, DMSO-d_6) δ 8.41 (d, *J* = 7.4 Hz, 1H), 8.12 (d, *J* = 6.6 Hz, 1H), 7.47 (d, *J* = 8.0 Hz, 2H), 7.39 – 7.30 (m, 3H), 5.06 – 5.00 (m, 2H), 4.54 (s, 2H), 4.33 – 4.27 (m, 1H), 4.26 – 4.20 (m, 1H), 4.18 (s, 1H), 3.89 – 3.84 (m, 1H), 3.57 (s, 3H), 2.38 – 2.25 (m, 2H), 2.08 – 2.02 (m, 1H), 1.98 – 1.92 (m, 1H), 1.79 – 1.68 (m, 1H), 1.22 (d, *J* = 7.1 Hz, 3H), 0.84 (dd, *J* = 29.6, 6.8 Hz, 6H). ^{13}C NMR (201 MHz, DMSO-d_6) δ 200.7, 173.2, 173.1, 171.4, 156.5, 138.4, 132.1, 128.1, 121.4, 83.7, 81.3, 65.2, 60.3, 56.1, 51.8, 48.6, 48.0, 40.4, 40.3, 30.7, 29.8, 24.9, 19.5, 18.5, 18.0. ESI-MS: *m/z* 522.2 $[M+H]^+$; HPLC t_R = 12.054 min (Column B), HPLC Purity: 95.2%.
